# Thermocatalytic epoxidation by cobalt sulfide inspired by the material's electrocatalytic activity for oxygen evolution reaction†

**DOI:** 10.1039/d4cy00518j

**Published:** 2024-07-16

**Authors:** Vanessa Wyss, Ionel Adrian Dinu, Laurent Marot, Cornelia G. Palivan, Murielle F. Delley

**Affiliations:** a Department of Chemistry, University of Basel 4058 Basel Switzerland murielle.delley@unibas.ch; b Department of Physics, University of Basel 4056 Basel Switzerland

## Abstract

New discoveries in catalysis by earth-abundant materials can be guided by leveraging knowledge across two sub-disciplines of heterogeneous catalysis: electrocatalysis and thermocatalysis. Cobalt sulfide has been reported to be a highly active electrocatalyst for the oxygen evolution reaction (OER). Under these oxidative conditions, cobalt sulfide forms oxidized surfaces that outperform directly prepared cobalt oxide in OER catalysis. We postulated that the catalytic activity of oxidized cobalt sulfide for OER could reflect a more general ability to catalyze O-transfer reactions. Herein, we show that cobalt sulfide (CoS_*x*_) indeed catalyzes the epoxidation of cyclooctene, a thermal O-transfer reaction. Similarly to OER, the surface-oxidized CoS_*x*_ formed under reaction conditions outperformed the directly prepared cobalt oxide, hydroxide, and oxyhydroxide for epoxidation catalysis. Another notable phenomenological parallel to OER was revealed by the electron paramagnetic resonance (EPR) analysis of all spent Co-based catalysts that showed significant structural changes and the formation of paramagnetic Co(ii) and Co(iv) species. Mechanistic investigations suggest that a higher density of Co(ii) and/or an easier formation of high-valent Co species in the case of surface-oxidized cobalt sulfide is responsible for its high activity as an epoxidation catalyst. Our results provide important insight into the surface chemistry of Co-based catalysts and show the potential of oxidized CoS_*x*_ as an earth-abundant catalyst for O-transfer reactivity beyond OER. This work highlights the utility of bridging electrocatalysis and thermocatalysis for the development of more sustainable chemical processes.

## Abstract 2

Cobalt sulfide catalyzes epoxidation reactions with parallels in the surface chemistry to electrocatalytic oxygen evolution reaction by the same material.

## Graphical abstract

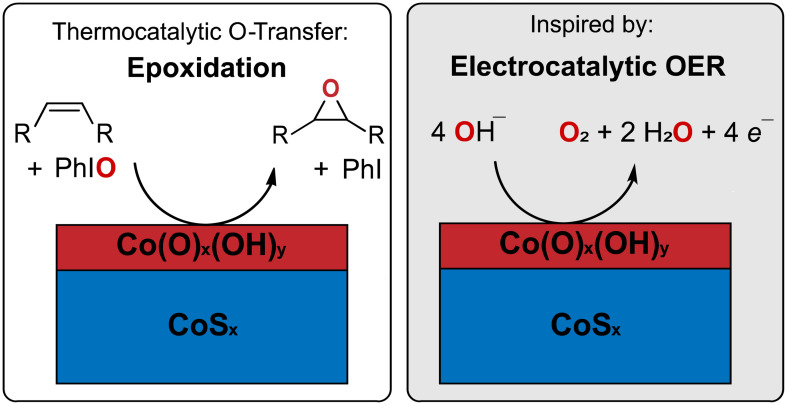

## Introduction

The development of more sustainable chemical processes depends on the design of better catalysts which incorporate earth-abundant materials. Catalyst design for heterogeneous systems has often relied on transferring known principles from enzymatic or molecular systems to solid-state catalysts.^1–5^ Another approach for catalyst design is to bridge the two sub-fields of heterogeneous catalysis, electrocatalysis and thermocatalysis.^6–12^ Many fundamental principles of catalysis are shared between the two fields. For instance, in both electro- and thermocatalysis, binding energies of key intermediates are often used as a descriptor to identify champion catalysts.^13,14^ Following this rationale, a catalyst with optimal binding energies for an intermediate that is critical for an electro- as well as a thermocatalytic reaction should show high catalytic activity in both reactions (though, considerations based on one single descriptor are unlikely to reflect the full complexity of heterogeneous catalysis^15,16^).^17^ For example, surface-adsorbed hydrogen is critical for electrocatalytic hydrogen evolution as well as for thermocatalytic hydrodesulfurization catalysis. It has been shown that MoS_2_ is a highly active catalyst for both reactions due to favorable adsorption properties for hydrogen on MoS_2_ edge sites.^18–21^ Similarly, the adsorption energy of oxygen (O*) has been identified as a descriptor of catalytic activity for both electrocatalytic CO_2_ reduction and thermocatalytic CO_2_ hydrogenation. This has led to the discovery of a new electrocatalytic system for CO_2_ reduction based on a well-studied Ni–Ga thermocatalyst.^22,23^ The different reaction conditions typically needed in electro- and thermocatalysis can impose different reaction kinetics and mechanisms, thus making it challenging to draw direct analogies between the two sub-fields. Nonetheless, the above precedents show that bridging concepts between heterogeneous electro- and thermocatalysis may provide new insight and inspire new discoveries in catalysis.

Connecting electro- and thermocatalysis is particularly useful if a large knowledge base built through a high research activity in one field can be leveraged. Oxygen evolution reaction (OER) is one example of an intensely researched electrocatalytic reaction for its relevance in the area of energy conversion and storage.^24–26^ While the OER mechanism is debated and depends on the particular catalyst used, it is generally accepted that it occurs by multiple proton and electron transfers and involves the binding of oxygen species to the electrode surface at an intermediate stage in the catalytic cycle.^27–29^ In a simplified view, OER could hence be regarded as an electrocatalytic O-transfer reaction. The binding energy of surface-adsorbed oxygen (O*) has been proposed as an activity descriptor for OER catalysts.^13,30^ This implies that the catalytic activity of different materials for OER correlates with an optimized ability to bind oxygen-based species intermediately. We hence propose that a catalyst active for OER having favorable binding energies for O* or related species, could also be a good catalyst for thermal O-transfer reactions, such as epoxidations. This hypothesis seems plausible in view of prior reports concerning manganese oxide OER catalysts used for electrocatalytic epoxidations,^31^ and molecular Fe complexes capable of catalyzing both OER and thermal epoxidations.^32,33^ Indeed, the Fe-based catalysis of OER and thermal epoxidation has been shown to occur *via* similar metal-oxido intermediates.^32,33^ This appears to support the idea that the two catalytic reactions can impose similar requirements for intermediate oxygen-binding.

Cobalt sulfide (CoS_*x*_) has shown promising catalytic properties for OER.^34,35^ At the high oxidative potentials required for OER, the pre-catalytic CoS_*x*_ oxidizes at the surface.^36–39^ This oxidized surface layer has been proposed to include Co_3_O_4_, CoOOH, Co(OH)_2_, or more generally, CoO_*x*_(OH)_*y*_ species.^37,38,40–42^ Interestingly, these oxidized CoS_*x*_ surfaces seem to catalyze OER more efficiently than the directly prepared cobalt oxide, oxyhydroxide, and hydroxide.^35,37,43^ This has variously been attributed to (i) the formation of larger surface areas, (ii) a higher conductivity due to a conductive sulfide particle core, (iii) the formation of surface structures that cannot be accessed by direct synthesis, (iv) synergistic interactions between the metal, S, and O components, or (v) an easier formation of high-valent Co (the presumed key intermediates for OER)^44–53^ in case of surface-oxidized transition metal sulfides.^37–43,54,55^ While the underlying cause remains unclear, these literature reports suggest that surface-oxidized CoS_*x*_ stands out among related Co-based OER catalysts and may hence also catalyze thermal O-transfer reactions.

In this report, we explore the use of CoS_*x*_ as a catalyst in a thermal O-transfer reaction. As a test O-transfer reaction, we selected the epoxidation of olefins, which are important chemical transformations to produce synthetically valuable epoxides.^56^ We have compared the catalytic performance of CoS_*x*_ to directly prepared cobalt oxide, oxyhydroxide, and hydroxide, and probed the formation of high-valent Co species and oxidized surface layers under epoxidation conditions by X-ray photoelectron spectroscopy (XPS) and electron paramagnetic resonance (EPR). We have also combined spectroscopic analysis and mechanistic investigations to obtain insight into the operating epoxidation mechanism. We discuss our observations in the context of the reported OER electrocatalysis by CoS_*x*_ to examine the utility of connecting thermocatalysis and electrocatalysis of O-transfer reactions.

## Experimental

### Hazard warning

Epoxidation reactions using PhIO as oxidant can lead to the formation of iodoxybenzene (PhIO_2_). This compound has been reported as potentially explosive and can decompose by violent combustion when heated under vacuum or scratched with a spatula. Decomposition temperature: 230 °C.^57,58^

### Chemicals

Cyclooctene (>95.0%), *cis*-2-octene (>95.0%) and *trans*-2-octene (>97.0%) were purchased from TCI. Thiourea (>99%), iodine (>99%), *tert*-butanol (>99%), sodium perborate tetrahydrate (96%), *tert*-butyl hydroperoxide (5.5 M in decane) and (diacetoxyiodo)benzene (98%) were purchased from Sigma Aldrich. *n*-Butyl lithium (2.5 M in hexane) (*n*-BuLi), perchloric acid (70% in water), 1,3,5-trimethoxybenzene (99%), thiophenol (99%) and cobalt acetate tetrahydrate (98–102%) were purchased from Acros Organics. Cobalt(ii)hydroxide (97%) (Co(OH)_2_) was purchased from Fisher Scientific. 4-*tert*-Butylphenol (97%) and 1-nitroso-2-naphthol-3,6-disulfonic acid, disodium salt hydrate (indicator grade) was purchased form Thermoscientific. 1-(*tert*-Butylsulfonyl)2-iodosylbenzene and iodosobenzene (PhIO) were synthesized according to reported literature procedures.^59,60^ H_2_O was purified using a Millipore water purification system equipped with a Quantum® TEX Polishing Cartridge. The purified water is referred to as MQ water below. EtOH (HPLC grade), Acetonitrile (MeCN) (HPLC grade), and CHCl_3_ (HPLC grade) were purchased from J.T. Baker. Reagent grade NaOH, NaCl, Na_2_SO_4_, NaHCO_3_, NH_4_Cl, acetic anhydride (99%), sulfuric acid (95%) (H_2_SO_4_), glacial acetic acid (100%), and hydrogen peroxide (35% in water) were purchased form VWR Chemicals. Et_2_O (stabilized) was purchased from Biosolve. All chemicals were used as received unless stated otherwise. Toluene was dried using a PS-MD-5 solvent purification system from Innovative Technologies, over activated alumina and Cu_2_O. Acetonitrile and chloroform were dried over 3 Å molecular sieves before use. Deuterated solvents were purchased from Apollo scientific, dried over 3 Å molecular sieves, and stored in an Ar-filled glovebox.

### General considerations

^1^H NMR spectra were recorded at 295 K on Bruker Avance III-400 or 500 NMR spectrometers. Gas chromatography-mass spectra (GC-MS) were recorded on a Shimadzu GCMS-2020 SE equipped with a Zebron 5 MS Inferno column (30 m × 0.25 mm × 0.25 mm). Samples for characterization by X-ray photoelectron spectroscopy (XPS) were transferred to the XPS instrument under inert atmosphere and measured in an ultra-high vacuum (UHV) chamber equipped with a monochromatic Al-Kα X-ray source (*hν* = 1486.6 eV) and a photoelectron spectroscopy analyzer (VG ESCALAB 210) with an energy resolution of 0.5 eV at 20 eV pass energy. Fitting was done using the Unifit program. The spectra were charge corrected by shifting of all the energy values in a way that the C 1s signal appears at the literature value of 284.8 eV for the amorphous carbon present in all measurements.^61^ Powder X-ray diffraction measurements were conducted on a STOE StadiP-powder-diffractometer with a Dectris Mythen 1K-detector and a micro-focused Cu-Kα-source (*λ* = 1.542 Å). Scanning electron microscopy (SEM) was done using a Zeiss Gemini2 microscope and processed on the SmartSEM User Interface. Energy dispersive X-ray (EDX) spectroscopy was done using the AMETEK EDAX Octane Elect EDS System. SEM and EDX measurements were performed at the Nano Imaging Lab, SNI, University of Basel. UV-vis spectra were measured on a Cary5000 UV-vis-NIR spectrometer by Agilent Technologies. Elemental analysis of all samples was conducted by the “Mikroanalytisches Labor Pascher” in 53424 Remagen, Germany. N_2_ adsorption measurements were conducted on an Autosorb IQ-X-MP-MP instrument by Quantachrome Instruments. Electron paramagnetic resonance (EPR) measurements were performed at room temperature with a X-band CW Elexsys-500 spectrometer equipped with a variable temperature unit. All samples were kept and measured under Ar atmosphere in Suprasil tubes. The EPR spectra were obtained as first derivatives and recorded with the following parameters: modulation frequency 100 kHz, microwave power 2 mW, number of scans 5–30, resolution 1024 points, modulation amplitude 5G and slow sweep.

### Material preparation

#### Preparation of cobalt oxide (Co_3_O_4_)

Co_3_O_4_ was prepared according to a reported hydrothermal method.^62–64^ Briefly, cobalt acetate tetrahydrate (250 mg, 1.0 mmol) was dissolved in 22 mL EtOH : H_2_O, 10 : 1. The solution was transferred into a 45 mL Teflon-lined stainless-steel Parr reactor and heated to 160 °C for 15 h. After allowing the mixture to cool to RT the black precipitate was centrifuged (rpm = 14 200, 5 min) and washed multiple times first with water and then with ethanol. The black powder was collected and dried under reduced pressure (5 × 10^−2^ mbar) yielding (25–50 mg).

#### Preparation of surface-oxidized cobalt sulfide (CoS_*x*_-ox)

CoS_*x*_-ox was prepared according to a reported hydrothermal method that was slightly adapted.^65^ Cobalt acetate tetrahydrate (250 mg, 1.0 mmol) and thiourea (75.0 mg, 1.0 mmol) were dissolved in 20 mL MQ water and stirred for 30 min. Then, the solution was transferred into a 45 mL Teflon-lined stainless-steel Parr reactor and the solution was heated to 180 °C for 24 h. The precipitate was centrifuged (rpm = 14 200, 5 min) and washed multiple times sequentially with water and ethanol. The black powder was collected and dried under reduced pressure (5 × 10^−2^ mbar) yielding (25–50 mg).

#### Preparation of cobalt sulfide (CoS_*x*_)

CoS_*x*_-ox (100 mg) was suspended in 7 mL degassed H_2_SO_4 (aq)_ (0.5 M) and sonicated for 5 min. Then, the solution was decanted from the sedimented cobalt sulfide (CoS_*x*_) and CoS_*x*_ was washed 3 times with MQ water (6 mL) and dried under reduced pressure (5 × 10^−2^ mbar) to give CoS_*x*_. A similar method has previously been reported to remove oxidized surface layers from cobalt phosphide.^63^

#### Preparation of PhIO-oxidized cobalt sulfide (CoS_*x*_-ox-PhIO)

CoS_*x*_ (32 mg) was suspended in 10 mL CHCl_3_ in a 25 mL Schlenk flask under inert atmosphere. Then, 1-(*tert*-butylsulfonyl)-2-iodosylbenzene (100 mg, 0.294 mmol) was added and the mixture was stirred for 16 h at RT. Then, the solvent was removed under reduced pressure and the residue was sequentially washed three times with CHCl_3_ and MeCN and dried under reduced pressure (5 × 10^−2^ mbar). A similar procedure has been followed to treat Co_3_O_4_, CoOOH, and Co(OH)_2_ with 1-(*tert*-butylsulfonyl)-2-iodosylbenzene.

#### Preparation of cobalt oxyhydroxide (CoOOH)

CoOOH was prepared according to a reported procedure.^66^ A dispersion of Co(OH)_2_ (100 mg, 1.08 mmol) in MQ water (20 mL) was heated to 45 °C. Then, NaOH_(aq)_ (8 M, 5 mL) and H_2_O_2_ (30%, 2 mL) were added together dropwise over 15 min. The mixture was stirred overnight for 16 h. The solvent was then removed by decantation and centrifugation and the brown precipitate was washed three times with MQ water before drying it under vacuum (5 × 10^−2^ mbar) yielding (80–95 mg).

### Catalytic epoxidation of cyclooctene by PhIO

CoS_*x*_ (10.0 mg, 0.11 mmol Co content assuming a Co_9_S_8_ composition), cyclooctene (0.65 mL, 5.00 mmol), and 1,3,5-trimethoxybenzene (5.00 mg, 0.18 mmol) as internal standard were added to a 5 mL Schlenk flask under N_2_-atmosphere equipped with a stir bar. Then, iodosobenzene (220 mg, 1.00 mmol) and toluene (1.5 mL) were added and the reaction mixture was stirred at 80 °C for 5 h. The reaction mixture was sampled at various time intervals (*t* = 0, 0.08 h, 0.25 h, 0.5 h, 1 h, and 5 h) and analyzed by ^1^H NMR. A similar procedure was followed for all materials examined herein (CoS_*x*_-ox, CoS_*x*_, Co_3_O_4_, Co(OH)_2_, and CoOOH) as well as for a control reaction in the absence of a material. Each experiment was replicated at least three times to assess reproducibility.

### Catalytic epoxidation of cyclooctene by ^*t*^BuOOH

CoS_*x*_ (10 mg, 0.11 mmol Co content assuming a Co_9_S_8_ composition), cyclooctene (0.65 mL, 5.00 mmol), toluene (1.5 mL) and *tert*-butyl hydroperoxide (^*t*^BuOOH) (0.2 mL, 1.00 mmol, 5.5 M in decane) were added to a 5 mL Schlenk flask under N_2_-atmosphere. The reaction was stirred at 80 °C for 5 h and was sampled at *t* = 0, *t* = 5 min and 5 h, and analyzed by ^1^H NMR using 1,3,5-trimethoxybenzene as an internal standard. A similar procedure was followed for all materials examined herein (CoS_*x*_-ox, CoS_*x*_, Co_3_O_4_, Co(OH)_2_, and CoOOH) as well as for a control reaction in the absence of a material.

### Influence of a radical scavenger on the epoxidation of cylooctene

The radical scavenger 4-*tert*-butylphenol was added after 30 min reaction time to one experiment of epoxidation of cyclooctene using either PhIO or ^*t*^BuOOH as oxidant and CoS_*x*_ as the catalyst material.

### Catalytic epoxidation of *cis*- and *trans*-2-octene with PhIO

CoS_*x*_ (10 mg, 0.11 mmol Co content assuming a Co_9_S_8_ composition), iodosobenzene (220 mg, 1.00 mmol) and 1,3,5-trimethoxybenzene (5.00 mg, 0.18 mmol) as internal standard were added to a 5 mL Schlenk flask under N_2_-atmosphere equipped with a stir bar. Then, *cis*- or *trans*-2-octene (0.5 mL, 3.25 mmol) and toluene (1.5 mL) were added. The reaction was stirred at 80 °C for 5 h and was sampled at *t* = 0 and 5 h and analyzed by ^1^H NMR. A similar procedure was followed for all materials examined herein (CoS_*x*_-ox, CoS_*x*_, Co_3_O_4_, Co(OH)_2_, and CoOOH) as well as for a control reaction in the absence of a material.

### Probing the isomerization of *cis*-2-octene

To test for the possibility of the catalysis of a *cis*/*trans* olefin isomerization by the Co-based materials during epoxidation catalysis, we exposed *cis*-2-octene to the different catalysts in the same solvent, temperature, and time scale as used for epoxidations (toluene, 80 °C, 5 h) and in absence of the oxidant. The reaction mixtures were each analyzed after 5 h of heating and stirring.

## Results

### I. Material syntheses and characterization

#### Preparation of cobalt-based materials

We prepared a series of cobalt-based materials: a cobalt sulfide that is oxidized at the surface (CoS_*x*_-ox), a cobalt sulfide that predominantly exposes the bare sulfide at the surface (CoS_*x*_), cobalt(ii,iii) oxide (Co_3_O_4_), cobalt(iii) oxyhydroxide (CoOOH), and cobalt(ii) hydroxide (Co(OH)_2_). CoS_*x*_-ox and Co_3_O_4_ were both synthesized in air by hydrothermal methods according to modified literature procedures, the former using cobalt(ii) acetate and thiourea in water at 180 °C and the latter using cobalt(ii) acetate in ethanol and water (10 : 1) at 160 °C.^62–65^ To obtain a cobalt sulfide that (mostly) exposes bare sulfide surfaces, CoS_*x*_-ox was treated with 0.5 M H_2_SO_4_ for 30 min to give CoS_*x*_. Similar procedures have previously been reported to remove oxidized layers from the surface of other cobalt-based inorganic materials.^63,64^ Commercially available Co(OH)_2_ was used as received. CoOOH was prepared by the reaction of Co(OH)_2_ with NaOH and H_2_O_2_ at 45 °C.^66^ See experimental for further details on material preparation.

#### Bulk composition, particle size, and surface area

**Fig. 1:**
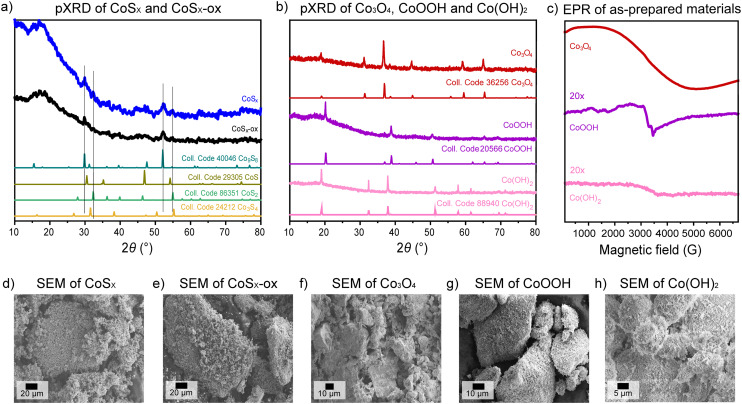
Bulk characterization of all as-prepared materials by (a and b) pXRD with comparison to literature diffraction pattern of different cobalt sulfide phases, Co_3_O_4_, CoOOH, or Co(OH)_2_. Reference pXRD patterns were obtained from the Inorganic Crystal Structure Database (ICSD). Analysis by (c) EPR, where the EPR spectra of CoOOH and Co(OH)_2_ have been vertically scaled to show minor signals, and by (d–h) SEM.

Elemental analyses, energy-dispersive X-ray spectroscopy (EDX), and powder X-ray diffraction (pXRD) combined gave insight on bulk composition and phase of the prepared CoS_*x*_-ox, CoS_*x*_, Co_3_O_4_, CoOOH, and Co(OH)_2_ materials. These analyses suggest that the obtained CoS_*x*_-ox and CoS_*x*_ materials were largely amorphous with average Co : S stoichiometries in the bulk of ∼1 : 1 and likely a small amount of crystalline Co_9_S_8_ and CoS_2_ phases present (Fig. 1a and ESI† sections S11 and S12). Bulk analyses of Co_3_O_4_, CoOOH, and Co(OH)_2_ suggested that the materials were partially amorphous, partially crystalline, and had average Co : O stoichiometries of ∼3 : 4, 1 : 2, and 1 : 2, consistent with cobalt(ii,iii) oxide, cobalt(iii) oxyhydroxide, and cobalt(ii) hydroxide phases, respectively (Fig. 1b and ESI† sections S11 and S12). Scanning electron microscopy (SEM) of the materials showed that all cobalt-based materials consisted of particles with a broad size distribution spanning roughly from few to 500 μm (Fig. 1d–h and S14†). N_2_ adsorption measurements were used to determine the surface areas of all Co-based materials. This method was selected over electrochemical methods of measuring surface areas as the former seems more relevant to the thermal catalysis examined herein. All Co-based materials exposed BET surface areas in the range of ∼20–40 m^2^ g^−1^ based on N_2_ adsorption experiments (Fig. S15†). The measured surface areas are relatively small consistent with bulk materials but larger than what would be expected for smooth particles of >1 μm. This hence suggests that the Co-based materials exhibit significant surface roughness on the order of 100–1000 (ESI† section S15). This is consistent with SEM showing nanostructured surfaces for all Co-based materials (Fig. 1d–h and S14). It is also possible that small particles that would not be detectable by SEM contribute to the measured surface areas.

EPR spectra were measured at room temperature and under non-saturating slow-passage conditions for all materials for insight on oxidation and spin states (Fig. 1c and S34†). However, the EPR cavity could not be tuned with samples of CoS_*x*_ and CoS_*x*_-ox (before catalysis), perhaps due to high sample polarity associated with dipolar interactions. The EPR spectrum of bare Co_3_O_4_ showed a very broad line (Δ*Γ* = 3900 G) with a *g*_eff_ ≈ 2.174 that can be attributed to tetrahedral Co(ii) (*S* = 3/2) in a spinel-type structure, consistent with prior reports.^67–69^ Commercial Co(OH)_2_ measured at room temperature had a low intensity EPR signal at *g*_eff_ ≈ 2.150 with a large line width (Δ*Γ* = 1570 G), possibly associated with low-spin Co(ii) species (*S* = 1/2). However, the very low intensity of the EPR signal prevents a clear assignment of the paramagnetic species. CoOOH only exhibited very low EPR signal intensity consistent with a majority of species being EPR-silent low-spin Co(iii). EPR analysis of CoOOH also reveals the presence of traces of high-spin Co(ii) species (*S* = 3/2) with *g*_eff_ ≈ 3.25 and (Δ*Γ* = 350 G), and of a free radical (*g* = 2.0024), perhaps originating from the synthesis involving H_2_O_2_.

#### Surface composition

**Fig. 2:**
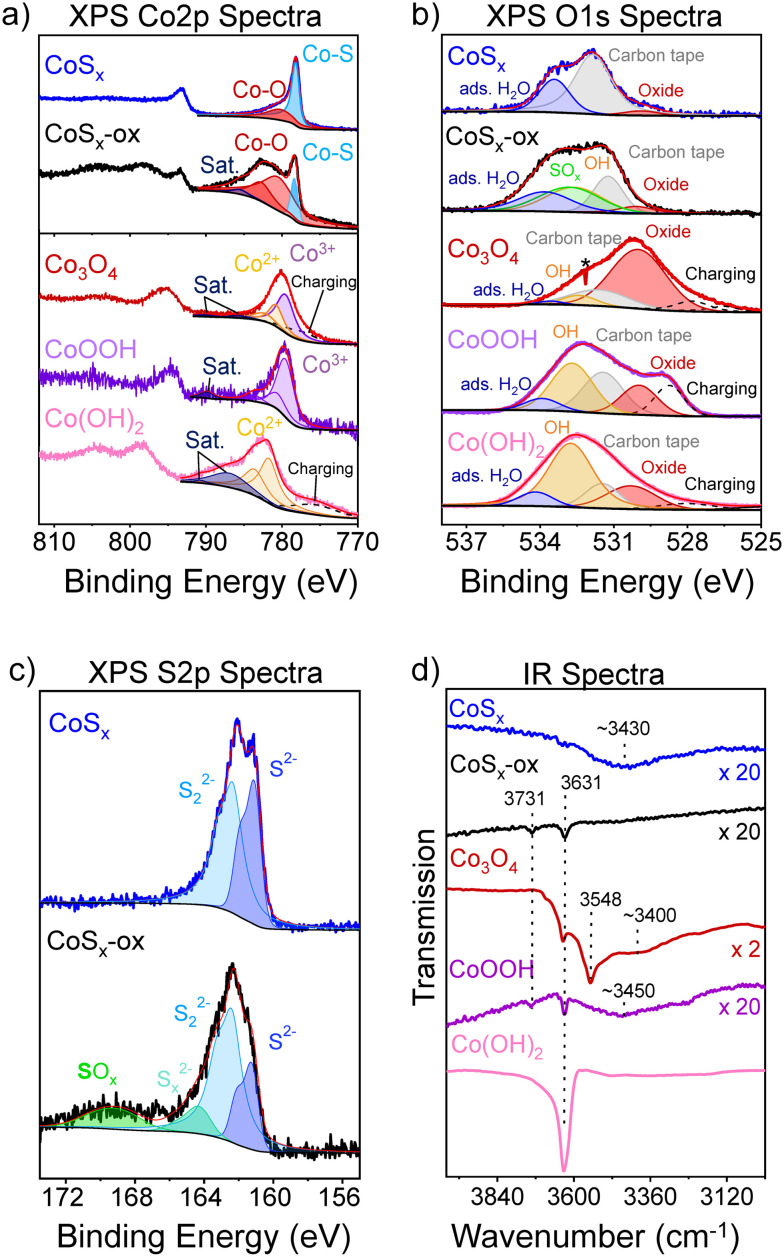
XPS Co 2p (a), O 1s (b), and S 2p spectra (c) and IR spectra (d) of CoS_*x*_ (blue), CoS_*x*_-ox (black), Co_3_O_4_ (red), CoOOH (purple), and Co(OH)_2_ (pink). Fitted components to the XPS spectra are shown as colored peaks. The asterisk in (b) denotes an instrumental artefact. Individual IR spectra have been vertically scaled as indicated on the right in (d) to show low intensity bands.

XPS and infrared (IR) spectroscopy provided insight into the surface composition of the as-prepared cobalt-based materials. In the currently known O-transfer catalysis by cobalt sulfides (the OER electrocatalysis), oxidized cobalt-sulfide surfaces are thought to be catalytically relevant.^34–43,70^ It is therefore of particular interest to assess the degree and composition of surface oxidation on the cobalt sulfides. We first examine fitted XPS data but note that slightly different XPS fits may also be reasonable. The Co 2p and S 2p XPS spectra of the as-prepared CoS_*x*_-ox showed signals at 778.4 eV and 161.3, 162.4 and 164.2 eV from cobalt sulfide (with the component at 164.2 eV perhaps corresponding to a polysulfide) (Fig. 2a and c).^71–76^ The component at 168.6 eV in the S2p spectrum can be attributed to oxidized sulfur species (SO_*x*_).^73–75^ We also identified at least two components of oxidized cobalt (“Co–O”) in the Co 2p spectra at 780.5 and 782.7 eV that may arise from two different chemical environments of Co, such as **Co**(SO_*x*_)_*y*_ and **Co**O_*x*_, or from multiplet splitting of cobalt species.^71–73,77^ A small component at 786.5 eV in the Co 2p spectra could correspond to a satellite line. Such satellites have previously been shown to be characteristic for Co(ii) and Co(iii) valence states in cobalt oxides and (oxy)hydroxides.^66,71,77,78^ However, a clear identification of the presence or absence of certain valence states in CoS_*x*_-ox from XPS data is not possible because the satellite line pattern associated with particular valence states may depend on the chemical environment of Co, which is different in sulfides than in oxides and (oxy)hydroxides.^79^ Fitting the O 1s XPS spectrum of CoS_*x*_-ox is consistent with the presence of at least five different O-based species, four of which could be attributed to S**O**_*x*_, Co**O**_*x*_, **O**H surface species, and adsorbed H_2_**O** based on reported XPS measurements of metal sulfates and of Co_3_O_4_, CoOOH, and Co(OH)_2_ (Fig. 2b).^66,74,75,78,80,81^ A fifth component probably corresponds to C**O**_*x*_ from the carbon tape used as a support in the XPS measurements (Fig. S18†). The presence of surface hydroxyl groups on CoS_*x*_-ox is supported by the IR spectrum of CoS_*x*_-ox showing weak bands at 3731 and 3631 cm^−1^ (Fig. 2d). Together, the data suggest CoS_*x*_-ox retained cobalt sulfide character but also presented oxidized species presumably from the preparation in air.

The XPS and IR results for CoS_*x*_ suggest that the oxidized surface of CoS_*x*_-ox has been mostly removed in CoS_*x*_ after acid treatment and that a predominantly bare cobalt sulfide surface is exposed: The Co 2p and S 2p XPS signals from **Co**–O and **S**O_*x*_ species vanished or decreased (Fig. 2a and c). The fitted O 1s spectrum is consistent with the absence of surface S**O**_*x*_ or **O**H species and the presence of adsorbed H_2_**O** with a small contribution from Co**O**_*x*_ (in addition to C**O**_*x*_ from the carbon tape) (Fig. 2b).^66,78,80,81^ Consistent with this XPS analysis, the IR spectrum of CoS_*x*_ did not show a band attributable to an OH group (unlike CoS_*x*_-ox), and only a broad band at ∼3430 cm^−1^ assigned to adsorbed H_2_O was observed (Fig. 2d). XPS analysis also suggested that the Co : S ratio at the surface changed upon washing with acid from 9 : 7 on CoS_*x*_-ox to a more sulfur-rich surface of Co : S 1 : 2 on CoS_*x*_ probably due to leaching of cobalt during the acid-washing procedure (ESI† section S10). Bulk analyses by elemental analysis and EDX suggested that the bulk Co : S ratio remained similar, *i.e.* roughly 1 : 1 (ESI† sections S11 and S12). Overall, these data showed that while a small amount of oxidized Co surface species remain, CoS_*x*_ mostly exposed bare cobalt sulfide with enriched sulfur content at the surface.

XPS data for Co_3_O_4_, CoOOH, and Co(OH)_2_ are generally consistent with the literature. However, the spectra are complicated by charging effects^82^ that broadened the signals and introduced additional signal intensity at low binding energies (Fig. 2a and b). The Co 2p spectra of Co_3_O_4_, CoOOH, and Co(OH)_2_ showed the main Co 2p_3/2_ peaks at 779.6, 779.6, and 781.7 eV, respectively, similarly to prior reports (Fig. 2a).^66,77,83–86^ The intense satellite line at 786.9 eV for Co(OH)_2_ is characteristic of Co^2+^ in cobalt oxides and (oxy)hydroxides, while the only weak satellite line at higher binding energies for CoOOH is characteristic of Co^3+^.^66,87^ Deconvolution of the O 1s spectra supported the presence of mainly metal-oxide “**O**^2−^” ions for Co_3_O_4_, both surface **O**H and “**O**^2−^” ions for CoOOH, and mainly surface **O**H for Co(OH)_2_, in addition to some adsorbed H_2_**O** for all materials (Fig. 2b).^66,84,86^ The IR spectrum of Co_3_O_4_ showed bands at 3631 and 3548 cm^−1^, and a broad band at ∼3400 cm^−1^ likely attributable to surface OH species and adsorbed H_2_O (Fig. 2d).^88–90^ The IR spectrum of Co_3_O_4_ also contains bands at ∼660 and ∼570 cm^−1^ consistent with Co–O vibrations of crystalline Co_3_O_4_, and bands between 1630 and 1300 cm^−1^ are assigned to residual acetate from the synthesis; this could not be removed by copious washing of the Co_3_O_4_ (Fig. S19†).^91^ The IR spectra of CoOOH and Co(OH)_2_ showed *ν̃*_OH_ bands and contributions from *ν̃*_Co–O_ bands.^88,92–94^ The combined XPS and IR data are therefore consistent with the formation of Co_3_O_4_, CoOOH, and Co(OH)_2_, and with surfaces that expose different types of hydroxyl groups.

### II. Catalytic epoxidation

**Fig. 3:**
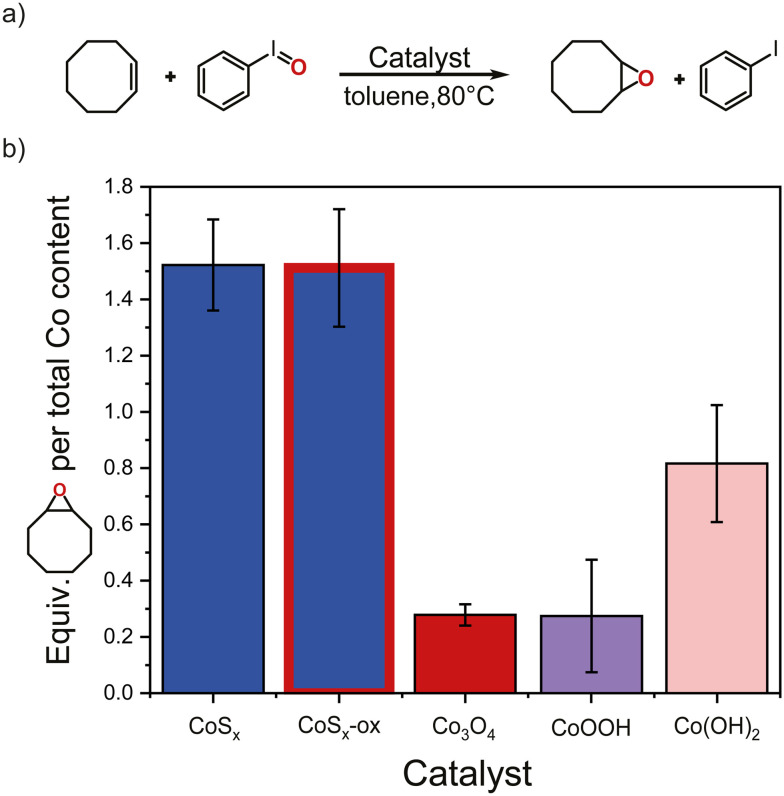
a) Thermal epoxidation of cyclooctene with PhIO using CoS_*x*_, CoS_*x*_-ox, Co_3_O_4_, CoOOH, or Co(OH)_2_ as catalyst. b) Amount of the formed cyclooctene oxide normalized with the *total* Co content in the catalyst material (CoS_*x*_, CoS_*x*_-ox, Co_3_O_4_, CoOOH, or Co(OH)_2_), here shown after 5 h reaction time. Error bars were determined from analysis of at least three independent replicate measurements. Actual TON of epoxidation are likely higher by a factor of ∼25–70 when taking into account the low fraction of exposed Co sites at the surface.

We tested the catalytic activity of the Co-based materials for thermal O-transfer reactions by probing alkene epoxidation reactions. Cobalt sulfides, Co(OH)_2_, and CoOOH have not, to the best of our knowledge, been previously tested for epoxidation catalysis, but the catalytic activity of Co_3_O_4_ and other Co-based materials for epoxidations have been reported.^95–104^ Cyclooctene was reacted with iodosobenzene (PhIO) in toluene at 80 °C in the presence of CoS_*x*_-ox, CoS_*x*_, Co_3_O_4_, CoOOH, or Co(OH)_2_, or in the absence of a catalyst (Fig. 3a). Each reaction was monitored by taking regular aliquots of the reaction mixture for quantitative ^1^H NMR spectroscopic analysis using an internal standard. All tested materials catalyzed the epoxidation of cyclooctene as indicated by the formation of cyclooctene oxide and iodobenzene (PhI); negligible cyclooctene oxide formation was observed in absence of Co-based materials (Fig. S1†). PhI was formed in large excess compared to cyclooctene oxide with all Co-based materials (Fig. S2†). Trace amounts of benzaldehyde (<0.01 mmol) (presumably formed from toluene) were also detected. However, this cannot account for the observed large excess of PhI with respect to cyclooctene oxide (Fig. S2†). A white solid formed during the reaction was identified by ^1^H NMR and pXRD (Fig. S6†) as PhIO_2_. This suggests that the excess of PhI is probably due to the decomposition of PhIO to PhI and PhIO_2_.^105,106^ This is also consistent with the observation of PhI but only negligible cyclooctene oxide in the absence of Co-based materials (Fig. S25†), and the observed fast formation of PhI compared to that of cyclooctene oxide for all Co-based materials (Fig. S2†). Due to the additional pathway of PhI formation, we take the amount of cyclooctene oxide formed normalized per *total* Co content in the material to compare *relative* catalytic activities among the different materials (Fig. 3b). Based on this analysis, the cobalt sulfides CoS_*x*_-ox and CoS_*x*_ had similar activity in cyclooctene epoxidation compared to each other and higher activity than Co_3_O_4_, CoOOH, or Co(OH)_2_ at 80 °C. Conducting cyclooctene epoxidation at room temperature suggested that CoS_*x*_-ox is more active than CoS_*x*_ showing lower activities by a factor of ∼4 and ∼7, respectively, compared to their respective activities at 80 °C (Fig. S4†). Since surface area can be an important factor for catalytic activity, we also compared cyclooctene oxide formation at 80 °C for the different materials normalized to surface area (Fig. S5†). However, since the surface areas of the Co-based materials changed under the reaction conditions (see below and Fig. S15†), it seems more appropriate to compare relative catalytic activities as in Fig. 3 using the total Co content in each material as normalization since this stays constant during catalysis (Fig. S12†). Nevertheless, similar qualitative conclusions are obtained from an analysis of cyclooctene oxide formation per initial or post-catalytic surface areas, namely that CoS_*x*_-ox and CoS_*x*_ outperformed Co_3_O_4_, CoOOH, and Co(OH)_2_ (Fig. S5†).

To obtain *absolute* measures of catalytic activity and turnover numbers (TONs) per active site for the different materials, the amount of cyclooctene oxide should be compared to the actual Co content *at the surface* for each material. However, it is difficult to obtain this number experimentally for the partially amorphous materials, which exhibit a large particle size and shape distribution. Based on surface areas and structures, we estimate that roughly 1–2% of the total Co content is on the surface (ESI† section S16). This corresponds to a TON of ∼90 for CoS_*x*_ and ∼110 CoS_*x*_-ox at 80 °C (Fig. S3†). It is likely that not all surface Co sites are catalytically active, hence TON for active sites will be even higher. With CoS_*x*_ and CoS_*x*_-ox we obtained a yield of cyclooctene oxide of ∼18% within 5 h based on the initial concentration of the oxidant added, while with Co(OH)_2_ ∼9%, and with Co_3_O_4_ and CoOOH only ∼3% yield was obtained (Fig. S1†). Catalyst performance in successive cycles is difficult to evaluate, because residual PhIO and PhIO_2_ cannot be separated from the spent catalyst. However, some insight might be gained from the addition of more PhIO after 1 h to the reaction mixtures with each Co-based material (Fig. S7†). These experiments suggest that after 1 h reaction time and upon addition of more PhIO, the cobalt sulfides are still highly active in epoxidation reactions and outperformed Co_3_O_4_, CoOOH, and Co(OH)_2_. The data also show that the product formation after the second addition of PhIO is roughly consistent with what is expected based on the surface area change of each Co-based material under reaction conditions, except for Co(OH)_2_, which seems to have lower activity per surface area after 1 h reaction time (ESI† section S3, Fig. S7). Overall, these data highlight the high catalytic ability for epoxidation of cobalt sulfides over Co_3_O_4_, CoOOH, and Co(OH)_2_.

We further tested the epoxidation of cyclooctene in the presence of each Co-based material using ^*t*^BuOOH instead of PhIO under otherwise similar conditions (toluene, 80 °C). Our aim was to see whether the catalytic ability of the Co-based materials for epoxidation of cyclooctene extended to other oxidants. All materials catalyzed the epoxidation of cyclooctene using ^*t*^BuOOH (Fig. S8†). The activity decreased in the series CoS_*x*_-ox ≅ CoS_*x*_ > Co_3_O_4_ ≅ Co(OH)_2_ > CoOOH, though CoS_*x*_-ox and CoS_*x*_ appeared to have an induction phase for catalysis at early reaction times. In contrast, when using PhIO as oxidant, the cobalt sulfides outperformed Co_3_O_4_, CoOOH, and Co(OH)_2_ throughout the course of the reaction (Fig. S1b†). Another difference with ^*t*^BuOOH was the observation of substantial amounts of allylic oxidation products (mostly 3-*tert*-butylperoxycyclooct-1-ene and traces of cyclooct-2-en-1-one) and oxidized toluene (mostly ((*tert*-butylperoxy)methyl)benzene and traces of benzyl alcohol and benzaldehyde) with all Co-based materials in addition to cyclooctene oxide (Fig. S9†). Allylic and benzylic oxidation products likely stem from H-abstraction by free radicals in solution (see Discussion).^107,108^ This complicates the analysis of the surface chemistry responsible for epoxidation reactions. Therefore, we focus below on the PhIO-based epoxidations that are largely selective for cyclooctene oxide formation.

#### Characterization of the spent catalysts

Post-catalytic examination of the Co-based materials after PhIO-based epoxidation is challenging due to the insolubility of PhIO and PhIO_2_ in most solvents. This prevents easy separation of the materials from the reaction mixture. Instead, we used a more soluble variant of PhIO to examine the materials after exposure to similar oxidative conditions; these are referred to as *e.g.* CoS_*x*_-ox-PhIO (see Experimental and ESI† section S8 for further details). Surface area measurements by N_2_ adsorption before and after exposure to the oxidative conditions showed that the surface areas of all materials changed under these conditions (Fig. S15†). While the surface area of CoS_*x*_ decreased significantly (from 38 to 25 m^2^ g^−1^), the surface areas of Co_3_O_4_ changed slightly (from 42 to 38 m^2^ g^−1^) and increased significantly for CoOOH and Co(OH)_2_ (from 33 to 43, and from 21 to 42 m^2^ g^−1^, respectively). Therefore, upon exposure to the oxidative conditions, the cobalt sulfides had a smaller surface area but produced larger amounts of product than Co_3_O_4_, CoOOH, and Co(OH)_2_.

**Fig. 4:**
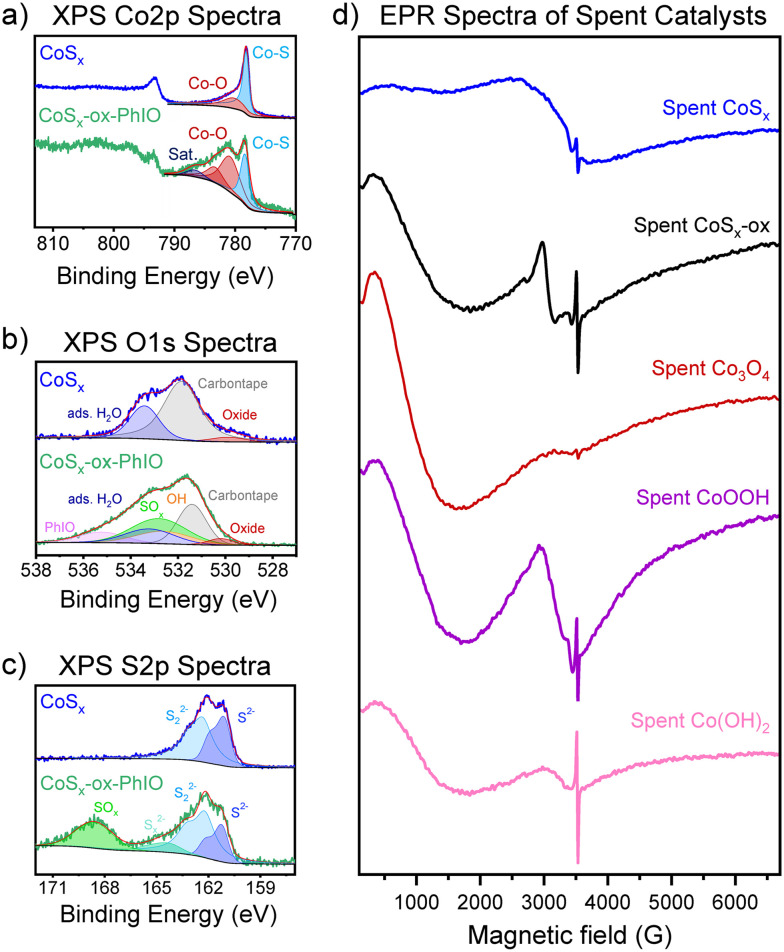
a) Co 2p, b) O 1s, and c) S 2p XPS spectra of CoS_*x*_ (blue) and CoS_*x*_-ox-PhIO (green). The XPS spectra of CoS_*x*_ are replicated from Fig. 2 for a more convenient comparison. d) EPR spectra of spent (post-catalytic) CoS_*x*_ (blue), CoS_*x*_-ox (black), Co_3_O_4_ (red), CoOOH (purple), and Co(OH)_2_ (pink).

The observed similar activities of CoS_*x*_-ox and CoS_*x*_ in the epoxidation of cyclooctene at 80 °C could be an indication that CoS_*x*_ and CoS_*x*_-ox expose similar surface structures during catalysis. Under the oxidative conditions employed for the epoxidation reactions, it is indeed expected that CoS_*x*_ is oxidized. XPS analysis of the CoS_*x*_ after exposure to the more soluble variant of PhIO (*i.e.*, CoS_*x*_-ox-PhIO) showed oxidized Co–O or Co(SO_*x*_)_*y*_ and SO_*x*_ surface species similar to that of CoS_*x*_-ox but with a higher amount of SO_*x*_ (Fig. 4a–c compared to Fig. 2a–c). The latter is probably due to the S-enrichment at the surface of CoS_*x*_ compared to CoS_*x*_-ox (see Results section I). The XPS data hence suggest that the surface of CoS_*x*_ was oxidized but also show that there is still cobalt sulfide content near the surface. We note that this XPS analysis does not allow identification of changes in Co valence states in CoS_*x*_, since it is not clear what binding energies and satellite line structures would be expected for different Co oxidation states in a cobalt sulfide. Potential shifts in binding energies after exposure to oxidizing conditions would also be difficult to interpret for Co_3_O_4_, CoOOH, and Co(OH)_2_ due to broad XPS signals and charging effects (see Results section I). This has hence not been attempted here. An observed color change of Co(OH)_2_ from pink to brown suggested that Co(OH)_2_ was also oxidized under epoxidation conditions. To investigate the oxidation of the catalytic materials under reaction conditions more directly, we probed all Co-based catalysts by EPR after catalysis and compared to the spectra of the bare materials.

The post-catalytic EPR analysis of the Co-based materials was performed in the presence of the residual PhIO_2_ and PhIO, which cannot be separated. This post-catalytic EPR analysis suggested that all Co-based catalysts changed significantly under reaction conditions (Fig. 4d compared to Fig. 1c and S34†). The EPR spectra of all Co-based materials after oxidative catalysis exhibited a broad signal at low magnetic field with *g*_eff_ ≈ 5.80–7.33 and (Δ*Γ* = 1370–1580 G) that was not observed for the bare materials. This intense first derivative signal can be associated with high-spin Co(ii) (*S* = 3/2) with octahedral symmetry.^109–112^ In the case of Co_3_O_4_, this signal is shifted to low magnetic field compared to the broad EPR line of the bare material associated with tetrahedral Co(ii) of the spinel (Fig. 1c). This suggests significant structural distortion of the spinel-type lattice of Co_3_O_4_. This is consistent with a prior report on Co_3_S_4_ nanosheets that had similar EPR signatures assigned to ferromagnetically coupled Co(ii) in a strongly distorted spinel-structure with change of the Co spin states.^113^

In addition to the EPR signal at low field, the EPR spectra of the spent CoS_*x*_, CoS_*x*_-ox, Co(OH)_2_, and CoOOH showed a second feature in the region of 2200–4500 G (Fig. 4d). The overall shape of this EPR feature indicates two superposed EPR patterns, which is more visible for CoS_*x*_-ox and CoOOH. This suggests the presence of two additional paramagnetic species. The extremely large line width of this superposed feature (Δ*Γ* = ∼1680 G) in the case of CoS_*x*_ makes a detailed assignment of these species difficult, but there appear to be at least two additional Co-based paramagnetic species, most likely a low-spin Co(iv) and possibly another Co(ii) species. For CoS_*x*_-ox, Co(OH)_2_, and CoOOH, one of the two species can be determined more precisely and has a *g*_eff_ of ∼2.2–2.4 and Δ*Γ* = 400–550 G. This is similar to the reported EPR signals obtained for CoO_*x*_ materials under OER conditions that have been assigned to low-spin Co(iv) (*S* = 1/2) species (potentially with some delocalization of the unpaired spin^114,115^).^109–112^ The formation of Co(iv) is generally consistent with the XPS Co 2p data shown in Fig. 4a for CoS_*x*_-ox-PhIO, as Co(iv) may contribute to the signals attributed to oxidized Co–O or Co(SO_*x*_)_*y*_ after exposure to the oxidizing conditions.^116^ However, it is not possible to unambiguously assign any particular XPS signal to Co(iv). Due to the large line width of the overall EPR pattern, a second minor component can be identified, for which gyromagnetic factors can be obtained by considering the wings of the EPR signal in the region of 2200–4500 G. This second species with a *g*_eff_ of roughly 3.4–3.6 can be tentatively assigned to a high-spin Co(ii) species with distorted geometry. However, variable temperature studies and high frequency EPR measurements are necessary to clearly assign the EPR *g* factors and the geometry for this second species. Interestingly, this minor second Co(ii) species at mid-field seems to qualitatively correlate with epoxidation activity: it is present for all the more active materials but not observed for Co_3_O_4_ (compare with Fig. 3). On the other hand, the major Co(ii) species at low-field and the Co(iv) species seem less likely to correspond to active species as these are also clearly visible for the least active epoxidation catalysts, Co_3_O_4_ and CoOOH. In addition to the features associated with Co, all spectra of the spent catalysts also exhibit a sharp signal with *g* = 2.0013–2.0024 that is characteristic for the presence of a free radical.

Our EPR results hence indicate that all Co-based catalysts reconstructed under the reaction conditions and formed multiple paramagnetic species including one or two high-spin Co(ii) species and in the case of CoS_*x*_, CoS_*x*_-ox, Co(OH)_2_, and CoOOH also a Co(iv) species. Interestingly, the paramagnetic Co-species formed under reaction conditions from the Co-based materials seem to be similar based on EPR despite different composition and oxidation states of the Co in the as-prepared materials. EPR also shows the formation of radicals under reaction conditions, associated with organic reaction products or perhaps inorganic radicals on the surface, such as O^−^.^117^

#### Mechanistic considerations

Epoxidation catalysis by ^*t*^BuOOH was inhibited in the presence of the radical scavenger 4-*tert*-butylphenol (Fig. S10b†). This demonstrated that free radicals are involved in the epoxidation catalysis. We also observed an inhibition of epoxidation catalysis when using PhIO and the radical scavenger (Fig. S10a†), and this is likely due to the reaction of PhIO itself with the radical scavenger.^107^ Clearer insight into the reaction pathway comes from an analysis of the reaction product distribution. The large amount of allylic and benzylic oxidation products from cyclooctene and toluene oxidation in the case of ^*t*^BuOOH-based epoxidation reactions is consistent with a free radical pathway. In contrast, no allylic oxidation products were observed in the case of PhIO. This suggests that the main epoxidation pathway does not involve free radicals but rather goes *via* a direct oxygen atom-transfer from PhIO to Co. The observations by NMR spectroscopy of trace amounts of benzaldehyde and by EPR of organic radicals suggest that radical or one-electron-transfer reactivity may occur in parallel.

Based on the similarity of the observed EPR features for all Co-based materials, and the observed correlation between the catalytic epoxidation activity (with PhIO) and a minor Co(ii) species, we speculate that this Co(ii) species may be involved in thermal epoxidation catalysis with all Co-based materials. Direct O-transfer from PhIO could lead to the formation of high-valent Co species, such as Co(iv)=O or Co(iii)–O·. The observation of Co(iv) by EPR demonstrates that the formation of such high-valent Co species is possible under reaction conditions. We note, however, that it is not necessarily this Co(iv) that is involved in epoxidation. A direct O-transfer would also be in line with the observed much faster formation of PhI than cyclooctene oxide (Fig. S2†). This suggests that the O-transfer from PhIO to the Co-based materials (and/or to PhIO in the PhIO decomposition pathway) is rapid. The formation of intermediate high-valent Co species would also be consistent with prior work on homogeneous Co-catalysts of the epoxidation of olefins using PhIO that involve similar Co-species.^107^

**Fig. 5:**
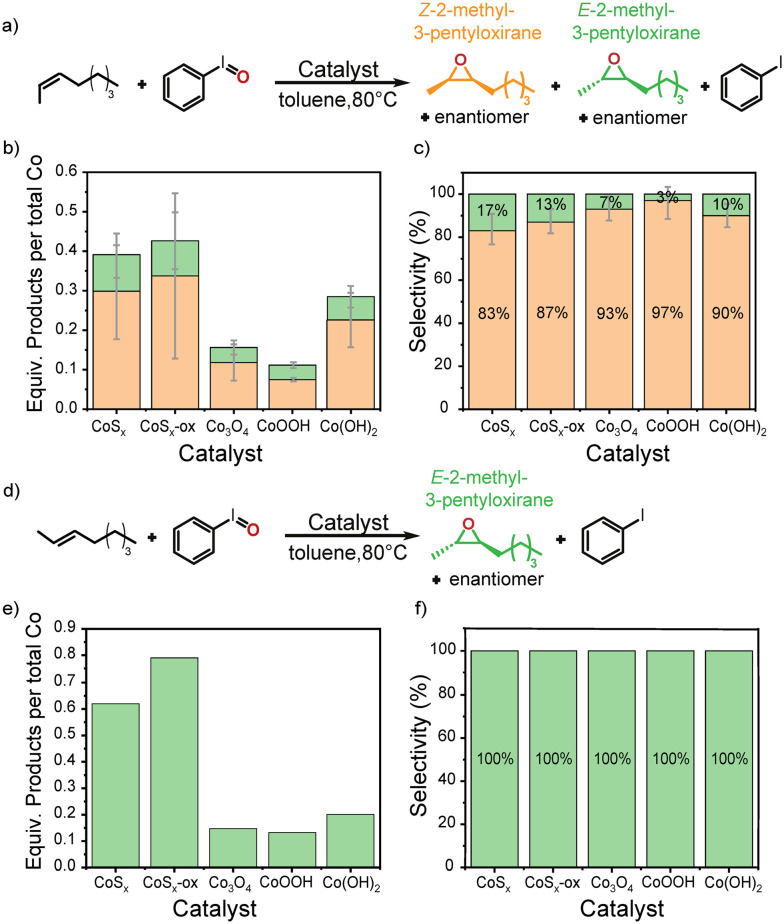
Thermal epoxidation of *cis*-2-octene (a–c) or *trans*-2-octene (d–f) with PhIO using CoS_*x*_, CoS_*x*_-ox, Co_3_O_4_, CoOOH, or Co(OH)_2_ as catalyst. (b and e) Show the amount of products normalized by the *total* Co content in each catalyst material after 5 h reaction time. Actual TON of epoxidation normalized to surface Co are likely higher by a factor of ∼25–70 when taking into account the low fraction of exposed Co sites at the surface. (c and f) Show the selectivity with which the *E*- or *Z*-epoxide is formed after subtraction of the amount of product formed from uncatalyzed reactions.

To obtain further mechanistic information into olefin epoxidation with PhIO using Co-based materials, we probed the epoxidation of *cis*-2-octene and *trans*-2-octene by PhIO in the presence of Co-based materials in toluene at 80 °C (Fig. 5). All Co-based materials were active catalysts for the epoxidation of *cis*-2-octene with relative activities decreasing in the series CoS_*x*_-ox ≅ CoS_*x*_ > Co(OH)_2_ > Co_3_O_4_ > CoOOH. However, there is a relatively large scatter in the data perhaps due to the lower reactivity of the linear olefins. Of particular relevance for mechanistic insight is the *cis*/*trans*-diastereochemistry of the epoxidation reactions. Compared to the uncatalyzed reaction that produced small amounts of both *E*-2-methyl-3-pentyloxirane and *Z*-2-methyl-3-pentyloxirane (*ca.* 64% and 36%, respectively, Fig. S11†), all Co-based materials significantly enhanced the level of stereoretention to the *Z*-epoxide but also showed stereoinversion. Subtracting the small amounts of both *E*- and *Z*-epoxide formed through the uncatalyzed epoxidation reaction reveals the changed product stereochemistry induced by the Co-based materials (Fig. 5c; Fig. S11† shows similar plots without subtraction). The Co-based materials catalyzed the epoxidation of *cis*-2-octene with 83–97% stereoretention and 3–17% stereoinversion. For the epoxidation of *trans*-2-octene only CoS_*x*_-ox and CoS_*x*_ showed significant catalytic activity, while barely any or no additional epoxide product was observed in presence of Co(OH)_2_, Co_3_O_4_, and CoOOH compared to the uncatalyzed reaction (Fig. 5e and S11c†). With *trans*-2-octene as substrate we only observed the *E*-epoxide as product consistent with a low driving force for stereoinversion.

## Discussion

### I. Catalysis and mechanism of thermal O-transfer reactions by Co-based materials

Our results demonstrate that cobalt sulfide is an active catalyst for the epoxidation of cyclooctene. To the best of our knowledge, this is the first report of the catalytic activity of a transition metal sulfide for epoxidation reactions. Epoxidation catalysis by Co_3_O_4_ has previously been reported.^95–97,101–103^ In this work, we have established that CoS_*x*_ and CoS_*x*_-ox outperformed Co_3_O_4_ by a factor of roughly five under the experimental conditions used. CoS_*x*_ and CoS_*x*_-ox can catalyze epoxidation reactions of both cyclic and linear olefins, already at room temperature, and with either PhIO or ^*t*^BuOOH as oxidants. This highlights the potential of CoS_*x*_ as a new material for the catalysis of epoxidation reactions.

**Scheme 1:**
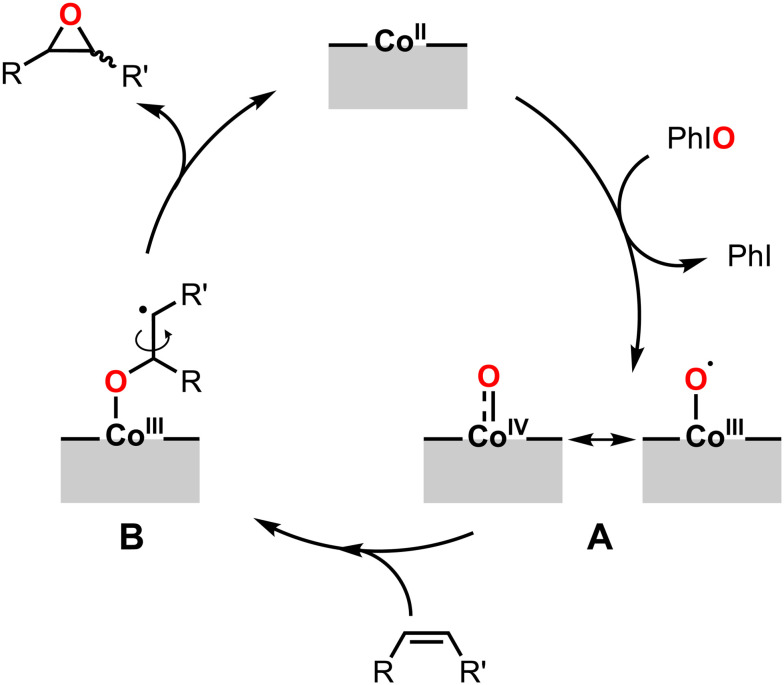
A plausible mechanism for the epoxidation of olefins with CoS_*x*_, CoS_*x*_-ox, Co_3_O_4_, CoOOH, and Co(OH)_2_.

The analysis of the spent catalysts combined with mechanistic investigations provided important clues about the mechanism of epoxidation using Co-based materials. It is interesting that our experiments showed similar paramagnetic species by the pseudo-*in situ* EPR measurements after PhIO-based epoxidations for all Co-based materials, as well as similar product distributions for all Co-based materials under different epoxidation conditions. These similarities suggest that epoxidation catalysis by the different Co-based materials occurs by similar reaction mechanisms. However, we expect some differences arising from the different chemical environments of the active Co sites in CoS_*x*_, CoS_*x*_-ox, Co_3_O_4_, CoOOH, and Co(OH)_2_. More detailed insight into these differences would require advanced *operando* investigations, but this is not possible with our current setup. Based on our results, we propose the following as a plausible reaction mechanism for the epoxidation with PhIO by the Co-based materials: (i) restructuring of all Co-based materials under reaction conditions forms new cobalt species, including an active Co(ii) species. (ii) Fast reaction of PhIO with Co(ii) could form high-valent Co(iv)-oxo or Co(iii)–O· species (**A**, Scheme 1). (iii) Based on the observed incomplete stereoretention in the epoxidation of *cis*-2-octene and since the Co-based materials do not catalyze olefin isomerization (see Experimental and ESI† section S7), coupling of the high-valent Co intermediate with an olefin could lead to an alkyl radical intermediate **B** that allows rotation around the C–C bond for linear substrates, followed by (iv) formation of the epoxide. Similar intermediates **B** have previously been proposed for homogeneous metal-complexes.^106,118^ The observed degrees of stereoinversion seemed to be higher for the S-containing CoS_*x*_ and CoS_*x*_-ox especially when compared to CoOOH, but also Co_3_O_4_ and Co(OH)_2_. This could be due to the extended lifetimes of **B** with the less electron-withdrawing S-content. The proposed mechanism involving Co(iii) or Co(iv) high-valent species also seems plausible in view of the high-valent Co intermediates that have typically been proposed for the epoxidation catalysis by molecular Co-complexes.^107,119–121^

With ^*t*^BuOOH, we observed substantial amounts of allylic and benzylic oxidation products during epoxidation catalysis, as well as a different initial relative activity pattern among Co-based materials compared to PhIO-based epoxidations. This probably reflects different mechanisms of epoxidation with the different oxidants. Koola and Kochi have previously suggested that such dual pathways for the epoxidation of olefins with PhIO or ^*t*^BuOOH are possible for homogeneous Co-catalysts.^107^ Both homogeneous and heterogeneous Co(ii)-based catalysts have been shown to initiate epoxidation catalysis with ^*t*^BuOOH by a Haber-Weiss initiation mechanism that involves an abstraction of HO· from ^*t*^BuOOH to form a Co(iii)–OH species, followed by H-abstraction from ^*t*^BuOOH to form Co(iii)–OO^*t*^Bu (Scheme S1†).^107,108,122,123^ Oxidation reactions are then thought to occur in solution by free radicals (Scheme S2†). This would be consistent with the observed product distribution and inhibition of catalysis with a radical scavenger. We speculate that Co(ii)/Co(iii) redox reactivity may also be responsible for the formation of the traces of benzaldehyde observed under PhIO-epoxidation conditions.

### II. Parallels to electrocatalytic OER

A particular goal of this study is to test whether leveraging knowledge from electrocatalysis could point towards activity for thermocatalysis by a given material. Indeed, the observed epoxidation catalysis by CoS_*x*_ is in line with our original proposal based on the material's reported electrocatalytic OER activity^34–43,70^ that CoS_*x*_ should be able to catalyze thermal O-transfer reactions. We further identify several interesting phenomenological parallels between the thermal and electrocatalytic O-transfer reactivity by CoS_*x*_:

(i) Relative activities. Similarly to what has been reported for Co-based OER catalysts,^35–41,43,70^ the cobalt sulfides showed higher activity for thermal O-transfer catalysis than directly prepared Co_3_O_4_, Co(OH)_2_, and CoOOH – even though CoS_*x*_ oxidized at the surface under epoxidation conditions. This seems to indicate that surface-oxidized cobalt sulfides have special abilities to catalyze O-transfer reactivity that go beyond OER. This is a key observation. Possible reasons for this distinctive ability are discussed below.

(ii) Surface oxidation and reconstruction. The observation that CoS_*x*_-ox exhibited slightly higher or similar catalytic activity for epoxidation than the bare CoS_*x*_ combined with the surface oxidation shown by the post-catalytic analysis of CoS_*x*_ by XPS and EPR suggest that oxidized cobalt sulfide surfaces are likely relevant under catalytic conditions, as in OER. The changes observed in color for Co(OH)_2_ and in BET surface area as well as XPS and EPR signatures for all spent Co-catalysts suggested that significant reconstruction of the material surfaces occurred under epoxidation conditions. For instance, the changes observed by EPR in the case of Co_3_O_4_ point towards significant structural distortion from the spinel-type structure to a structure containing (pseudo-)octahedral Co(ii). It has been suggested that this type of structural rearrangement also occurs for Co_3_O_4_ under OER conditions and has been connected to OER activity.^44,47,124,125^ It is interesting that all Co-based materials exhibited similar EPR signals associated with octahedral Co(ii) after catalysis, and most of the spent materials also showed similar features corresponding to two further paramagnetic Co-species. This is particularly surprising as the *as-prepared* Co-based materials differ significantly in composition, bulk structures, cobalt oxidation states, and morphology and degree of crystallinity. However, these observations are reminiscent of the behavior of Co-based materials under OER conditions: cobalt sulfide, oxide, hydroxide, as well as oxyhydroxide OER (pre-)catalysts all undergo reconstruction and form similar structural motifs (*i.e.*, amorphous CoO_*x*_(OH)_*y*_) at the surface under the oxidative reaction conditions.^38,40,44,45,124,126–128^

(iii) Populations of paramagnetic Co(ii) and Co(iv). A notable phenomenological parallel between the reported OER and the thermal epoxidation catalysis by Co-based materials studied here comes from our observation of EPR signals corresponding to populations of both Co(ii) and Co(iv) species. This is strikingly similar to the EPR evidence for Co(ii) and Co(iv) populations that have previously been reported for Co-Pi water oxidation catalysis.^109,115^

These phenomenological parallels (i–iii) raise the question as to whether thermal epoxidation and electrocatalytic OER by Co-based materials are related on a more fundamental level. Comparison of the mechanism and kinetics of the reported reaction pathways of OER^24–29,44–53,111^ and our proposed pathways of epoxidation uncovers mostly differences. This is expected because of the very different substrates, products, and reaction conditions needed for the two chemical transformations. These substantial differences limit the extent to which analogies can be made. Nevertheless, some parallels exist in Co redox chemistry that is relevant for both chemical transformations. The mechanism we propose in Schemes 1 and S1† starts with Co(ii) and involves high-valent cobalt intermediates: Co(iv)=O or Co(iii)–O· with PhIO, and Co–OO^*t*^Bu when using ^*t*^BuOOH as oxidant. Similarly, OER is usually thought to proceed through Co(ii)/Co(iii) and Co(iii)/Co(iv) redox couples,^44,45,47–53,109^ and Co(iv)=O/Co(iii)–O· and Co(iii)–OOH as key intermediates.^38,40–42,129,130^ These similarities in the underlying Co redox chemistry are significant, because they appear to be connected to the origin of the observed higher activity of cobalt sulfides over corresponding oxides and (oxy)hydroxides, as discussed below.

While surface-oxidized CoS_*x*_ had a higher activity than Co_3_O_4_, Co(OH)_2_, and CoOOH, the materials exhibited similar features by EPR and similar stereoretention properties in epoxidation catalysis of linear olefins. In addition, the cobalt sulfides had smaller post-catalytic surface areas than Co_3_O_4_, Co(OH)_2_, or CoOOH. All combined implies that either the density of active Co(ii) on the surface is different for different materials or that the rate-determining step of epoxidation comes before intermediate **B** and before the selectivity determining step (*i.e.*, the epoxide formation from **B**) in the catalytic cycle (Scheme 1). *We hence speculate that CoS*_*x*_*-ox either has a higher density of active Co(ii) species and/or the structure and properties of the S- and O-containing surface in CoS*_*x*_*-ox are responsible for an easier formation of high-valent Co intermediates **A** and **B***, perhaps through a less electron-withdrawing character from the S-content. This conclusion parallels prior proposals made for the observed high activity of surface-oxidized cobalt sulfides for OER.^35,40,41,43,131^ Effects from the density of active Co(ii) would explain the observed slightly lower activity of CoS_*x*_ for epoxidation that has S-enriched (Co-depleted) surfaces but larger surface area in the as-prepared state compared to CoS_*x*_-ox. Easier formation of high-valent intermediates due to a less electron-withdrawing character from the S-content would be consistent with the slightly higher stereoinversion observed in epoxidations with the cobalt sulfides. In addition, in view of the observed XPS signals characteristic for cobalt sulfide (in addition to oxidized components) after exposure to epoxidation conditions, it is conceivable that the S-content near the surface influences catalysis. For OER, other proposals have also been made to explain the high catalytic performance of oxidized transition metal sulfides over related oxides, such as a higher surface area, a conductivity of the surface-oxidized CoS_*x*_ particles due to a conductive sulfide core, or the formation of unusual amorphous or metastable phases that are difficult to gain by conventional synthesis.^37,38,43,54^ However, these cannot explain the differences in thermal epoxidation catalysis studied herein. Surface areas are smaller for the cobalt sulfides. The formation of amorphous surfaces does not seem to be exclusive to cobalt sulfides, as the formation of amorphous Co(O)_*x*_(OH)_*y*_ surfaces from cobalt oxides under OER conditions is well-documented.^44,45,124,126,128,132,133^ Furthermore, the conductivity of the catalyst material is unlikely to be of primary relevance for thermal reactivity. It is hence most likely that structural or electronic properties related to the S, O, and Co-containing surface in surface-oxidized cobalt sulfides lead to a higher density of active Co(ii) sites or an easier formation of high-valent Co intermediates and that this is responsible for the higher catalytic activity of these materials over related oxides and (oxy)hydroxides. It is notable that this seems to be the case for both thermal and electrocatalytic O-transfer reactions and hence could reflect fundamental properties of Co-based materials that are relevant to both OER and thermal epoxidations.

## Conclusion

We report the catalytic activity of cobalt sulfide for epoxidation of olefins, a thermal O-transfer reaction. This discovery was inspired by the previously reported electrocatalytic O-transfer activity by CoS_*x*_ for OER.^34–43,54,55^ We have identified several parallels between the thermal O-transfer catalysis by the cobalt-based materials and electrocatalytic OER: (i) cobalt sulfide outperformed the directly prepared Co_3_O_4_, Co(OH)_2_, and CoOOH, (ii) cobalt sulfide oxidized at the surface, (iii) all Co-based materials underwent significant structural changes under reaction conditions, and (iv) similar paramagnetic Co(ii) and Co(iv) species were formed with all Co-based materials. Characterization of the spent Co-based catalysts combined with mechanistic investigations suggested the involvement of high-valent Co(iv) or Co(iii) intermediates in Co-based epoxidations that are similar to those of OER, and a radical alkyl intermediate that forms the epoxide with competitive rates as C–C bond rotation. Similarly to what has been proposed for OER, we speculate that the higher activity of surface-oxidized cobalt sulfides compared to Co_3_O_4_, Co(OH)_2_, and CoOOH for epoxidation catalysis is due to a higher density of active Co(ii) sites and/or an easier formation of the key high-valent Co intermediates.

Our study highlights the utility of leveraging knowledge across the heterogeneous thermocatalytic and electrocatalytic communities for new catalytic discoveries and provides new perspectives on the surface chemistry and the O-transfer reactivity of cobalt-based materials. A picture emerges for cobalt sulfide catalysts that emphasizes their ability to catalyze O-transfers spanning both electrocatalytic and thermocatalytic reactions and to accommodate structural change and the formation of Co(ii) and Co(iv) species. Our work suggests that OER catalysts should be logical candidates when screening materials for thermal epoxidation catalysis. Further investigation will show whether these considerations extend to other O-transfer reactivity and if taking inspiration from CoS_*x*_ or other highly active OER-catalysts could point to earth-abundant catalyst materials that could complement or perhaps even replace current noble-metal based epoxidation processes.

## Data availability

The data supporting this article have been included as part of the ESI.†

## Author contributions

V. W.: investigation, methodology, data analysis, visualization, writing – original draft, review & editing; I. A. D.: investigation (EPR), visualization (EPR), writing – review & editing; L. M.: methodology (XPS), data analysis (XPS), writing – review & editing; C. G. P.: methodology (EPR), data analysis (EPR), supervision, writing – review & editing; M. F. D.: conceptualization, methodology, data analysis, visualization, funding acquisition, project administration, supervision, writing – original draft, review & editing.

## Conflicts of interest

There are no conflicts to declare.

## Supplementary Material

CY-014-D4CY00518J-s001
