# Supplementary material for: Thermocatalytic epoxidation by cobalt sulfide inspired by the material's electrocatalytic activity for oxygen evolution reaction
Source: Catal Sci Technol. 2024 Jul 16;14(16):4550–65. doi: 10.1039/d4cy00518j (PMC11318377; doi:10.1039/d4cy00518j)
Supplement: CY-014-D4CY00518J-s001 [file CY-014-D4CY00518J-s001.pdf]

Electronic Supplementary Material (ESI) for Catalysis Science & Technology.
This journal is © The Royal Society of Chemistry 2024

Supporting Information for Thermocatalytic Epoxidation by Cobalt Sulfide Inspired by the Material's Electrocatalytic Activity for Oxygen Evolution Reaction

Vanessa Wyss,[†] Ionel Adrian Dinu,[†] Laurent Marot,[‡] Cornelia G. Palivan,[†] Murielle F. Delley[†,*]
[†] Department of Chemistry, University of Basel, 4058 Basel, Switzerland
[‡] Department of Physics, University of Basel, 4056 Basel, Switzerland
murielle.delley@unibas.ch

Table of Content

1 Catalytic epoxidation of cyclooctene by PhIO S2
2 $PhIO_2$ formation S5
3 Successive catalytic cycles S6
4 Catalytic epoxidation of cyclooctene by ^{t}BuOOH S7
5 Influence of a radical scavenger on the epoxidation of cyclooctene S8
6 Catalytic epoxidation of *cis*- and *trans*-2-octene with PhIO S9
7 Probing the isomerization of *cis*-2-octene S10
8 Cobalt sulfide materials after exposure to oxidative conditions that are similar to those of epoxidation reactions S11
9 Assessing a potential leaching of cobalt into the reaction mixture by UV-Vis spectroscopy S12
10 UV-Vis spectra of acid washing solutions from the preparation of CoS_x S13
11 Elemental analysis data S14
12 Energy-dispersive X-ray spectroscopy (EDX) data S15
13 Scanning electron microscopy (SEM) images S16
14 N_2 adsorption measurement results S17
15 Estimated surface roughness S18
16 Estimated fraction of cobalt atoms at the surface S19
17 X-ray photoelectron spectroscopy (XPS) data S20
18 Infrared (IR) spectroscopy data S22
19 NMR spectroscopy data from the epoxidation of cyclooctene by PhIO S23
20 NMR spectroscopy data from the epoxidation of cyclooctene by ^{t}BuOOH S26
21 NMR spectroscopy data from the epoxidation of *trans*-2-octene by PhIO S32
22 NMR spectroscopy data from the epoxidation of *cis*-2-octene by PhIO S33
23 Electron paramagnetic resonance (EPR) spectroscopy data S34
24 Plausible mechanism of the epoxidation of cyclooctene by ^{t}BuOOH S35
25 References S36

1 Catalytic epoxidation of cyclooctene by PhIO

To compare the catalytic activity of different materials the amount of product produced was normalized to the total amount of cobalt atoms (in mmol) in the catalytic material, as shown in Fig. 3 in the main text. In Fig. S1 we show the absolute amount of product formed to compare the reaction in presence of the different catalytic materials to the control reaction in the absence of a material. Fig. S2 shows the amount of benzaldehyde and PhI formed in presence of the different catalytic materials. Fig. S3 provides estimated TONs. Fig. S4 shows the catalytic activity of cobalt sulfides at RT compared to their catalytic activity at 80 °C. Fig. S5 shows the amount of product formed normalized with initial and post-catalytic surface areas for the different materials.

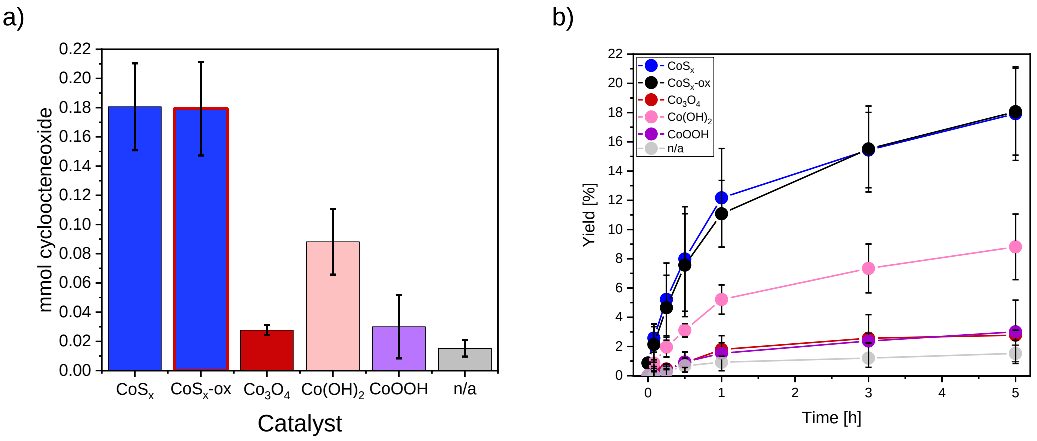

Fig. S1. a) Absolute amount of the formed cyclooctene oxide using CoS_x, CoS_x-ox, Co_3O_4, $Co(OH)_2$, or CoOOH, or in the absence of a catalyst after 5 h reaction time. Here we show absolute amount of product formed to compare catalytic performance of the different materials versus the reaction in absence of a material. In the main text we show a similar figure normalized to the total Co content in the reaction mixture to compare different catalysts with each other (Fig. 3 in the main text). b) Yield of cyclooctene oxide in % based on the initial concentration of the oxidant PhIO added, plotted versus reaction time

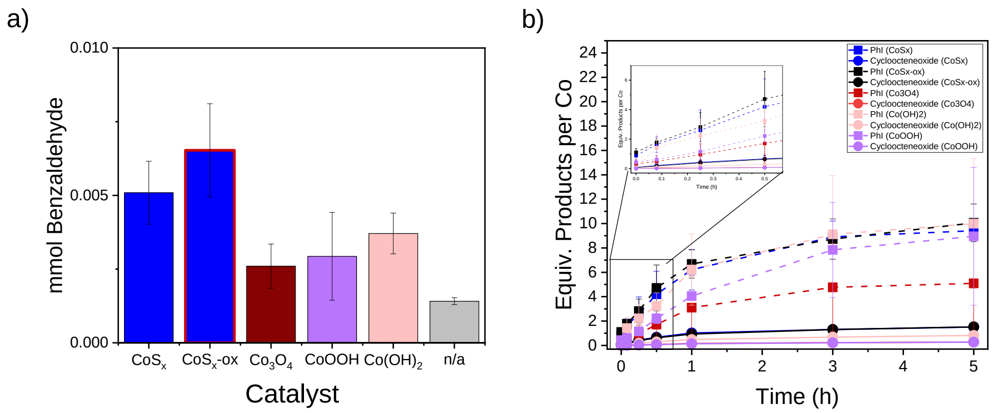

Fig. S2. Products formed during the epoxidation of cyclooctene using Co-based materials. a) Absolute amount of benzaldehyde formed from the solvent toluene after 5 h reaction time using different Co-based materials. b) Formation of iodobenzene (PhI; dashed lines) and cyclooctene oxide (solid lines) normalized with respect to the total Co content in the reaction mixture for each catalyst material over time. The large excess of PhI observed compared to the amount of cyclooctene oxide formed is likely due to the also occurring thermal decomposition of PhIO to PhI and $PhIO_2$, which is catalyzed by the cobalt-based materials.

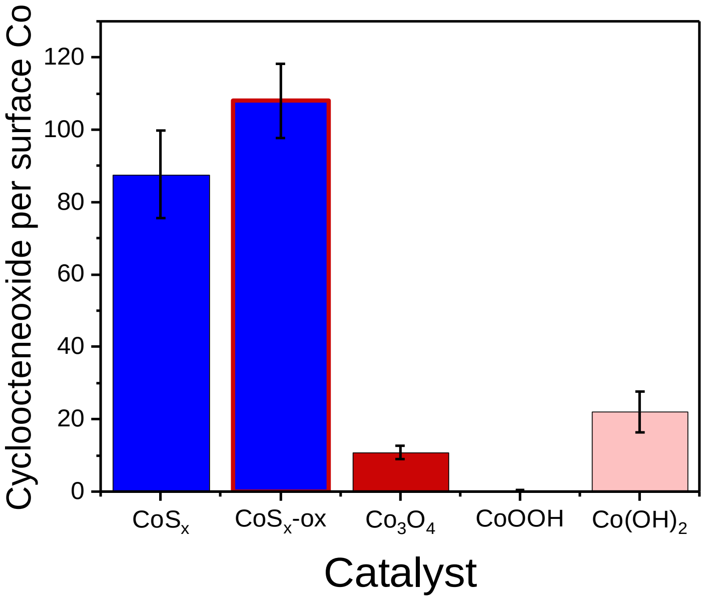

Fig. S3. Estimated TON after 5 h reaction time for different Co-based materials from the amount of cyclooctene oxide formed and a normalization to a roughly estimated amount of cobalt exposed at the surface. The amount of surface cobalt was estimated via the measured BET surface area for the different materials and assuming uniform particle sizes, spherical particle shapes and exposure of certain crystal facets (see SI section S16 for further details). Due to the inherent assumptions to these estimates the thereby obtained TON likely provide correct orders of magnitude but should not be taken as accurate absolute values.

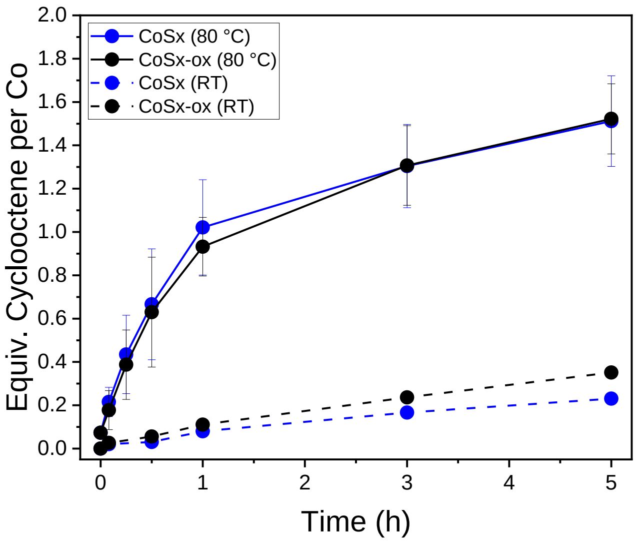

Fig. S4. Amount of cyclooctene oxide formed normalized with respect to the total Co content in the reaction mixture using CoS_x or CoS_x-ox in the epoxidation of cyclooctene with PhIO at RT (dashed lines) and at 80°C (solid lines).

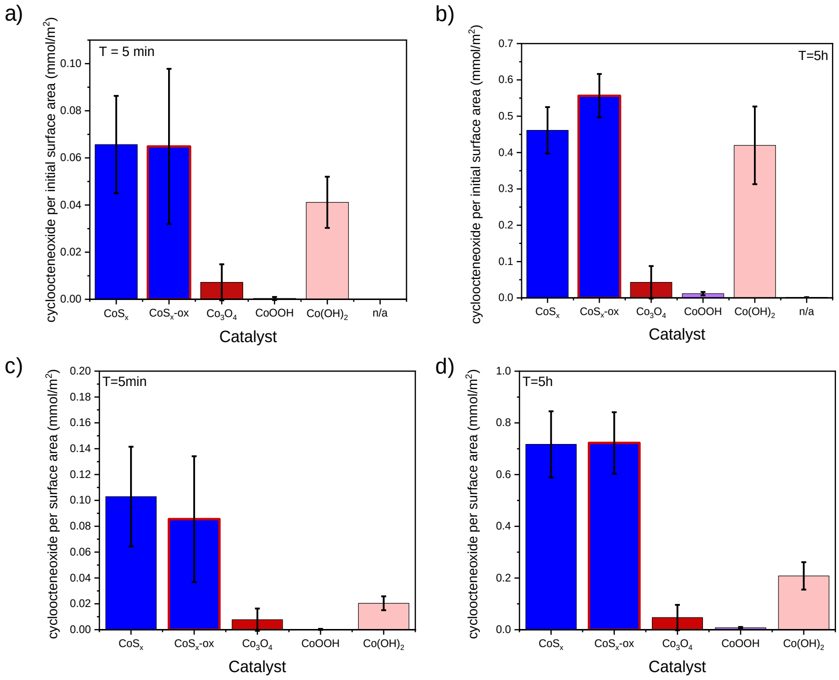

Fig. S5. Amount of cyclooctene oxide formed in the epoxidation of cyclooctene with PhIO using different Co-based materials normalized to the surface area of the as-prepared materials (Fig. S15) after (a) 5 min and (b) 5 h reaction time; and amount of cyclooctene oxide formed normalized by the surface area obtained after exposure to oxidative reaction conditions (Fig. S15) after (c) 5 min and (d) 5 h reaction time. These data show that similar qualitative conclusions are obtained by normalization to surface area as by normalization to total Co content, as discussed in the main text: CoS_x and CoS_x-ox outperform Co_3O_4, CoOOH, and $Co(OH)_2$ in the epoxidation of cyclooctene using PhIO.

2 $PhIO_2$ formation

Thermal decomposition of PhIO during the epoxidation reaction led to the formation of PhI and $PhIO_2$. This decomposition was accelerated in presence of (was catalyzed by) the herein examined Co-based materials. We recovered a white solid (mixtures with Co-based materials look grey) from the epoxidation reactions in presence and absence of Co-based materials and identified the solid as $PhIO_2$ by pXRD and NMR spectroscopy (Fig. S6). Please also see the **hazard warning** for $PhIO_2$ in the experimental section of the main text.

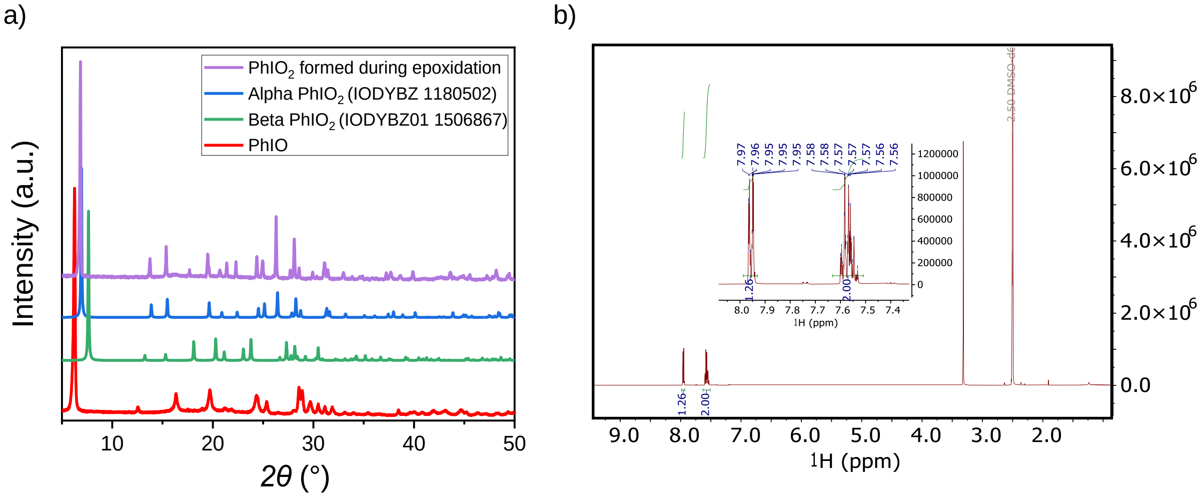

Fig. S6. a) pXRD patterns of the recovered $PhIO_2$ (mixed with CoS_x-ox) from the epoxidation of cyclooctene with PhIO (purple), the as-synthesized PhIO used for catalyis (red), and the computed patterns of alpha $PhIO_2$ (blue) and beta $PhIO_2$ (green) retrieved from the Cambridge Crystallographic Data Centre (CCDC) Crystallographic Database accessed on 07.03.2024. The comparison shows that the recovered solid formed during the reaction is distinct from the used PhIO and can be identified as predominantly alpha $PhIO_2$. b) ^{1}H NMR (500 MHz, DMSO-d_6) spectrum of the recovered $PhIO_2$ showing δ/ppm: 8.01 – 7.92 (m, 2H), 7.62 – 7.51 (m, 3H). Comparison to literature spectra is consistent with the identity of the white solid being $PhIO_2$.[1]

3 Successive catalytic cycles

Catalyst performance in successive cycles is difficult to evaluate for the catalytic systems used herein, because PhIO and $PhIO_2$ cannot be separated from the spent catalyst. For some insight on the activity of the Co-based materials after exposure to catalytic conditions, we added more PhIO (220 mg, 1.00 mmol together with 0.5 mL of a solution of 3.3 mM cyclooctene and 0.02 mM 1,3,5-trimethoxybenzene in 1.5 mL toluene) to the reaction mixtures with each Co-based material after 1 h reaction time in one experiment. Fig. S7 shows the increase in product formation after addition of more PhIO at t = 1h compared to the product formation versus time under our standard catalytic conditions. To infer on each Co-based material's activity after exposure to catalytic conditions, we compared the product formation observed during the first hour of reaction (t = 0 to t = 1h) to the product formation observed during the first hour after addition of more PhIO (t = 1h to t = 2h). Based on this analysis, the catalytic activity of CoS_x for epoxidation has decreased by ~25 %. This activity decrease is roughly consistent with or a bit less than what would be expected based on the surface area decrease of CoS_x upon exposure to oxidative conditions of ~34% (see Fig. S15). CoS_x-ox showed a similar decrease in product formation after further addition of PhIO by ~30 %. By a similar analysis Co_3O_4 showed increased product formation of ~18 % after addition of more PhIO, which is roughly consistent or a bit higher than what is expected based on a relatively constant surface area (Fig. S15). CoOOH showed an increased product formation by ~43 %, which is roughly consistent or a bit smaller than what is expected based on the surface area increase by 53% (Fig. S15). $Co(OH)_2$, on the other hand, showed a decreased product formation after addition of more PhIO by 67%, despite an increase of surface area under oxidative conditions by a factor of two (Fig. S15).

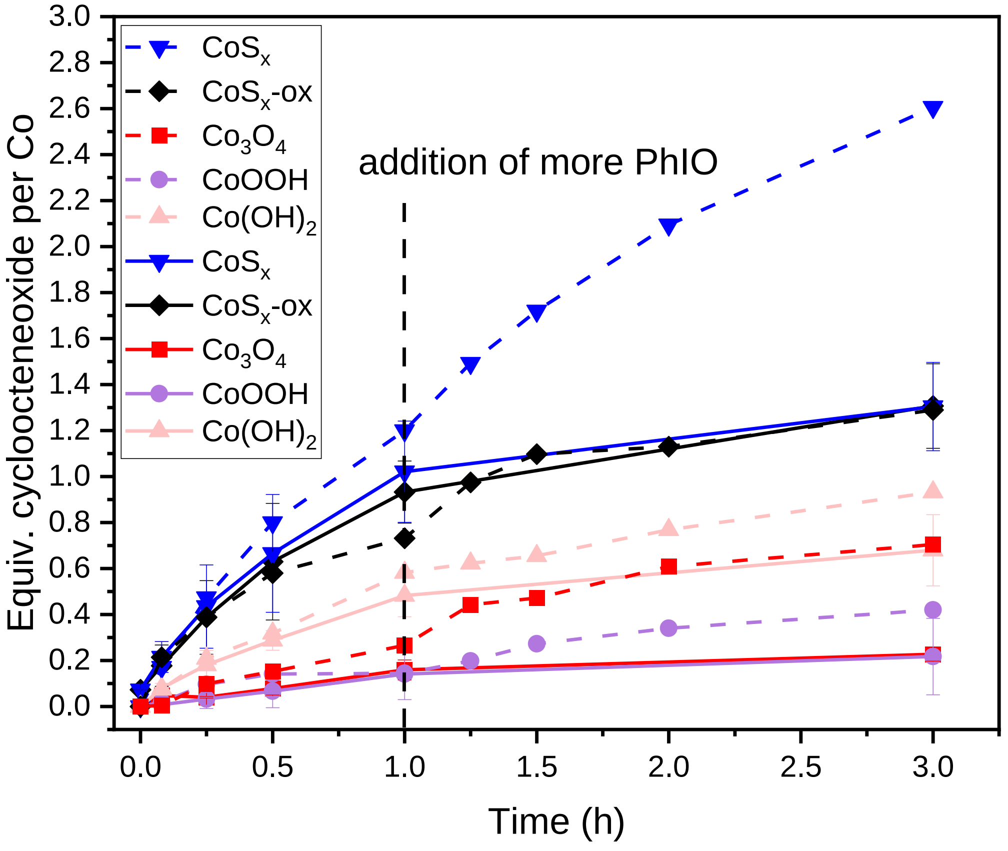

Fig. S7. Amount of cyclooctene formed with respect to total amount of Co in the material versus time from at least three replicate experiments (solid lines) is compared to experiments, where after 1 h reaction time more PhIO has been added (dotted lines). The data of the solid lines at 5 h reaction time is shown in the main text, Fig. 3.

4 Catalytic epoxidation of cyclooctene by ^{t}BuOOH

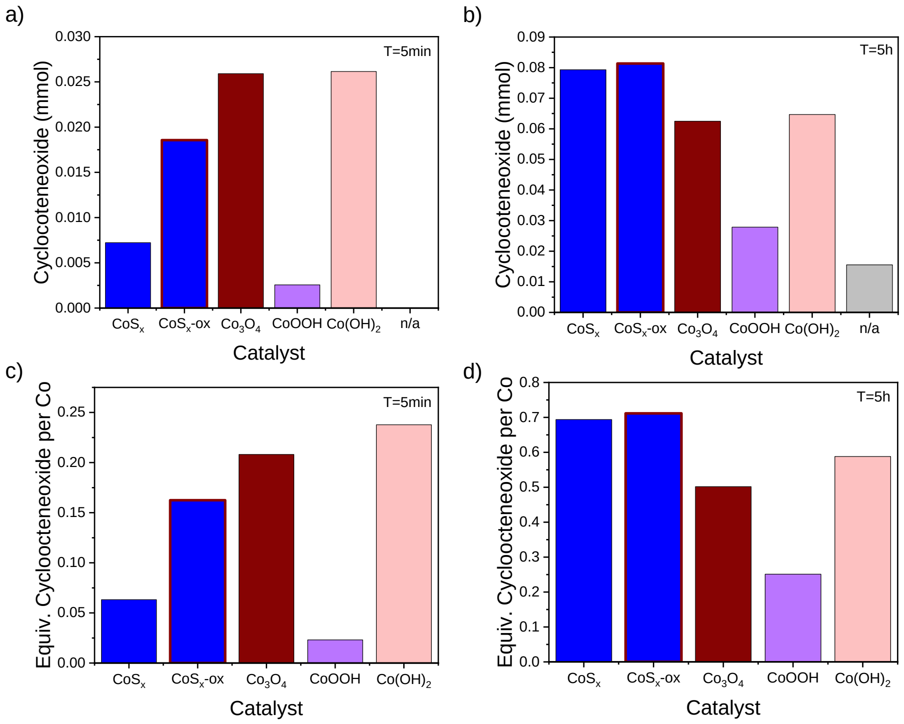

Fig. S8. Absolute amount of cyclooctene oxide in mmol formed in the epoxidation of cyclooctene using ^{t}BuOOH after (a) 5 min and (b) 5 h reaction time for each Co-based material and in the absence of a catalyst. The amount of cyclooctene oxide formed normalized to the total cobalt content in each material after (c) 5 min and (d) 5 h reaction time.

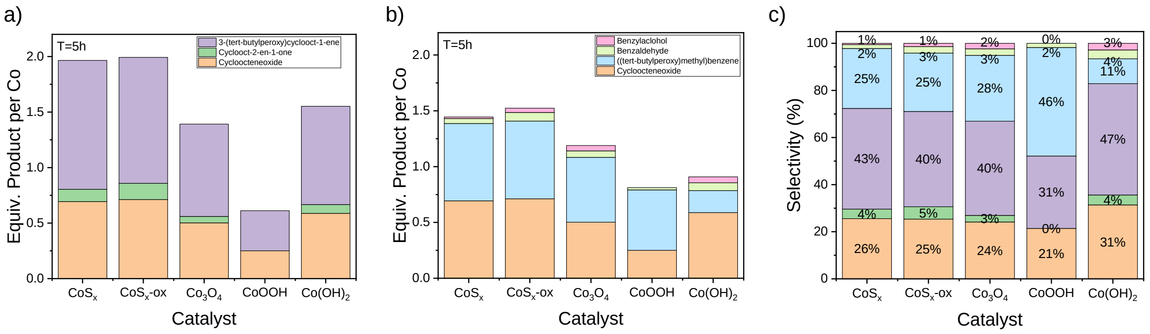

Fig. S9. Reaction products formed after 5 h reaction time in the epoxidation catalysis using ^{t}BuOOH in presence of different Co-based materials normalized to the total Co content in the mixture: (a) Products obtained from the oxidation of cyclooctene, and (b) products obtained from the oxidation of the solvent toluene. c) Selectivity of all reaction products for each material. With all Co-based materials 3-*tert*-butylperoxycyclooct-1-ene, cyclooct-2-en-1-one, ((*tert*-butylperoxy)methyl)benzene, benzyl alcohol, and benzaldehyde were observed in addition to cyclooctene oxide, except no cyclooct-2-en-1-one with CoOOH, which exhibited the lowest activity overall.

5 Influence of a radical scavenger on the epoxidation of cyclooctene

While for ^{t}BuOOH the observed inhibition of catalysis is consistent with a free radical mechanism, the radical scavenger can also directly react with PhIO,[2] and the observed inhibition of catalysis is likely not indicative of a radical mechanism in case of PhIO.

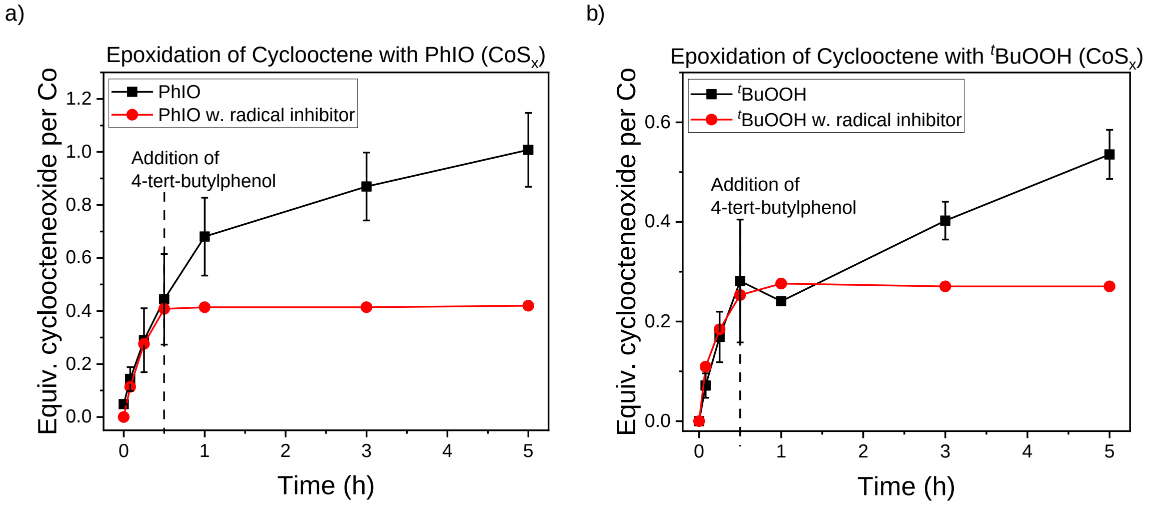

Fig. S10. Cyclooctene oxide formation during the epoxidation of cyclooctene normalized to the total amount of cobalt in the catalyst material (black) and with addition of radical inhibitor 4-*tert*-butylphenol at t = 30 min (red) using (a) PhIO and (b) ^{t}BuOOH as the oxidant.

6 Catalytic epoxidation of *cis*- and *trans*-2-octene with PhIO

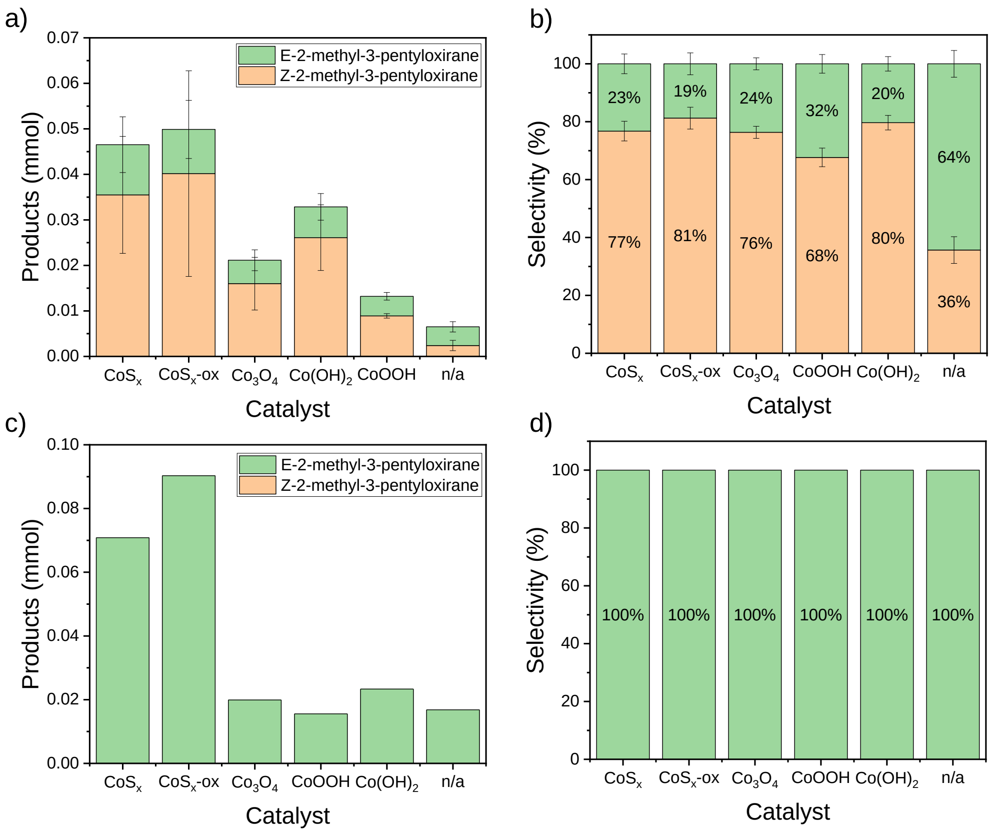

Fig. S11. Thermal epoxidation of *cis*-2-octene (a,b) or *trans*-2-octene (c,d) with PhIO using CoS_x, CoS_x-ox, Co_3O_4, CoOOH, or $Co(OH)_2$ as catalyst. (a,c) show the absolute amount of products formed (*Z*-2-methyl-3-pentyloxirane (orange) and *E*-2-methyl-3-pentyloxirane (green)) after 5 h reaction time in order to compare the catalytic performance of the different materials to the uncatalyzed reaction in the absence of a material. In the main text we show a similar figure normalized to the total Co content in the reaction mixture to compare different catalysts with each other (Fig. 5 in the main text). (b,d) show the selectivity with which the *E*- or *Z*-epoxide are formed for different materials without first subtracting the amount of products formed in the uncatalyzed reaction. Similar plots with such a subtraction are shown in Fig. 5 in the main text.

7 Probing the isomerization of *cis*-2-octene

The commercially available *cis*-2-octene contained trace amounts of *trans*-2-octene, but this amount stayed constant after 5 h and no increase or decrease in the ratio of *cis*:*trans* olefin (as would be expected in case of isomerization catalysis) could be observed with any of the tested catalysts nor in absence of a Co-based material. Hence, thermal isomerization of *cis*-2-octene is not catalyzed by the Co-based materials and does not occur. Isomerization of *cis*-2-octene can therefore not explain the formation of *E*-2-methyl-3-pentyloxirane observed in the epoxidation of *cis*-2-octene. We also note that the trace amount of *trans*-2-octene present in commercially available *cis*-2-octene does not change during epoxidation catalysis and is likely not the source of the observed *E*-2-methyl-3-pentyloxirane.

8 Cobalt sulfide materials after exposure to oxidative conditions that are similar to those of epoxidation reactions

Due to the insolubility of PhIO in most solvents the catalytic materials are difficult to separate from the epoxidation reaction mixtures. To analyze the materials after exposure to oxidative conditions that are similar to those of the epoxidation catalysis, CoS_x was treated with a soluble alternative of PhIO, namely 1-(*tert*-butylsulfonyl)2-iodosylbenzene, to give CoS_x-ox-PhIO (see main text, experimental section). The XPS data of CoS_x-ox-PhIO showed similar features as the XPS spectra of CoS_x-ox (see main text, Figs. 2 and 4) indicating that the CoS_x surface was oxidized when treated with 1-(*tert*-butylsulfonyl)2-iodosylbenzene. Please note that the chemical nature of the CoS_x-ox-PhIO surface after reaction with 1-(*tert*-butylsulfonyl)2-iodosylbenzene can only provide an approximation of what happens under actual epoxidation reaction conditions with PhIO, i.e. CoS_x-ox-PhIO is perhaps not identical to the materials after epoxidation catalysis using PhIO. For instance, in contrast to reactions of alkenes with PhIO in the presence of the cobalt sulfides, similar reactions using 1-(*tert*-butylsulfonyl)2-iodosylbenzene as the oxidant did not lead to epoxide product formation.

9 Assessing a potential leaching of cobalt into the reaction mixture by UV-Vis spectroscopy

After completion of the catalytic epoxidation reactions with PhIO using the different Co-based materials, each reaction mixture was filtered and the solvent was removed. Then, 3 mL of an aqueous solution of disodium 3-hydroxy-4-nitrosonaphthalene-2,7-disulfonate (Nitroso-R-salt) (0.5 mM, in MQ water) was added to each reaction mixture and the resulting solutions were diluted by a factor of 10 with MQ water. UV-Vis spectra were then recorded of each mixture and compared to a 0.05 mM solution of the Nitroso-R-salt in MQ water and to a 0.05 mM solution of Nitroso-R-Salt in MQ water also containing 0.005 mM $Co(OAc)_2•4H_2O$ as a representative sample of a solution containing Co(II)-ions (Fig. S12). The Nitroso-R-salt forms a complex with cobalt which has an absorption maximum at 395 nm and can be best identified (distinguished from the absorption of the free Nitroso-R-salt) by the absorption at 500 nm.[3] Co(II) can be identified in the last solution, in which $Co(OAc)_2•4H_2O$ has been added on purpose. None of the reaction mixtures from the epoxidation catalysis with different Co-based materials showed an absorption characteristic of cobalt suggesting that no cobalt leaches from the catalyst or that leaching occurs only in quantities below the detection limit. Based on our control with $Co(OAc)_2•4H_2O$ we would be able to detect the leaching of 0.1 % of the cobalt content in the catalysts used (typically 0.11 mmol cobalt) and probably much less.

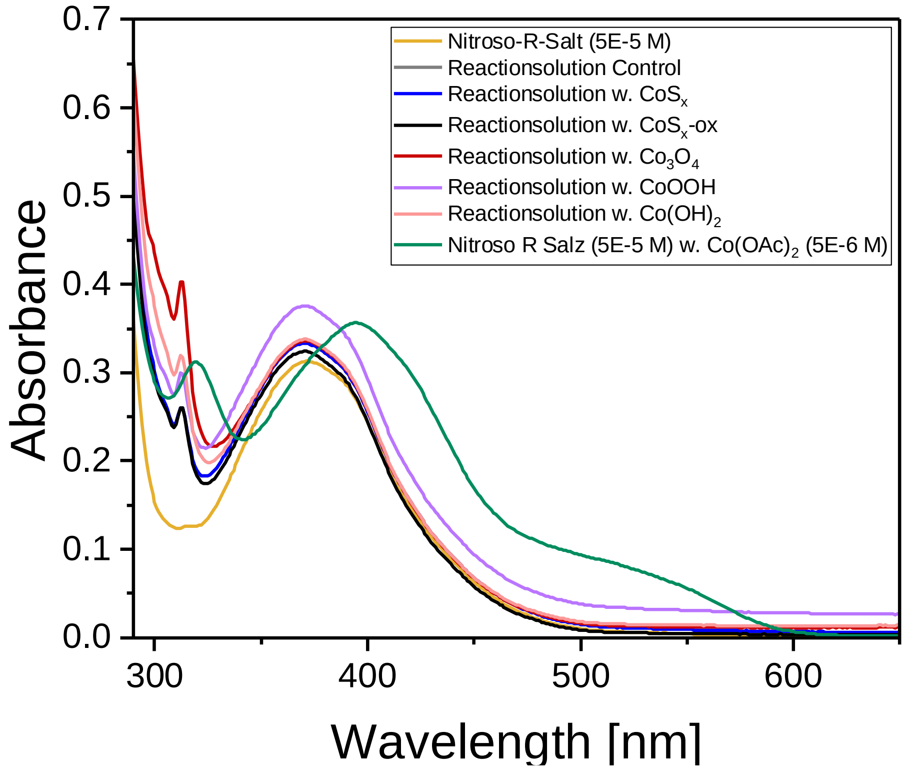

Fig. S12. UV-Vis spectra of the filtered and dried reaction mixtures after epoxidation catalysis using different Co-based materials in a 0.05 mM solution of Nitroso-R-Salt in MQ water and comparison to a pure 0.05 mM solution of Nitroso-R-Salt in MQ water, and a 0.05 mM solution of Nitroso-R-Salt in MQ water also containing 0.005 mM $Co(OAc)_2•4H_2O$.

10 UV-Vis spectra of acid washing solutions from the preparation of CoS_x

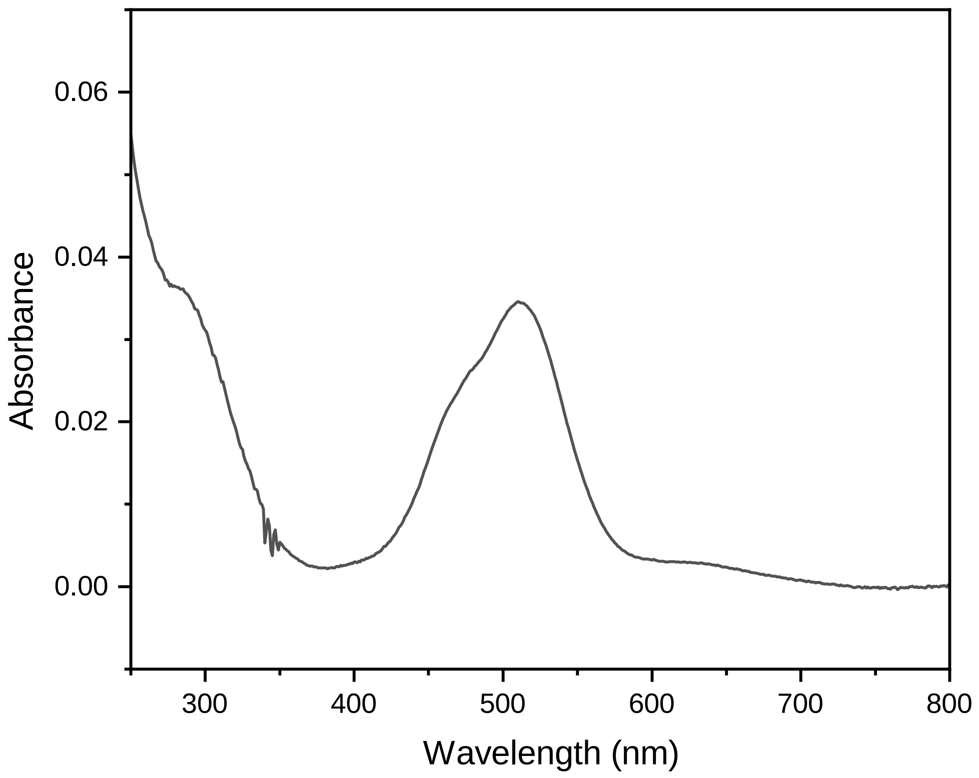

Fig. S13. UV-Vis spectrum of a washing solution of the acid treatment of CoS_x-ox, shown for a representative batch of the prepared CoS_x. The maximum absorbance at 512 nm was compared to UV-Vis spectra of solutions of cobalt acetate ($2.5\cdot10^{-3}$ to $5\cdot10^{-2}$ mM) in 0.5 M H_2SO_4 used as calibration. This analysis and similar UV/Vis data obtained for other batches of CoS_x prepared under similar conditions suggest a removal of typically ~0.03-0.07 mmol of cobalt from the surface of CoS_x-ox accounting for roughly 3 to 6 % of the total amount of cobalt content in the material. The sharp features at ca. 350 nm are caused by an instrumental artifact.

11 Elemental analysis data

Table S1. Elemental analysis results for CoS_x-ox, CoS_x, Co_3O_4, $Co(OH)_2$, and CoOOH. Each elemental analysis was performed twice. The measured mass percentages of the as-prepared materials suggest elemental Co:S ratios of 9:10 for both CoS_x-ox and CoS_x, and Co:O of 3:4, 1:2, and 1:2, for Co_3O_4, $Co(OH)_2$, and CoOOH, respectively.

Sample	Element	Result mass-% Measurement 1	Result (mass-%) Measurement 2	Mean (mass-%)	Stoichiometry
CoS_x-ox	Co	59.2	58.9	59.1	9
	S	32.7	35.3	34.0	10
	O	6.11	6.05	6.08	3
CoS_x	Co	58.7	57.8	58.3	9
	S	36.5	36.3	36.4	10
	O	5.46	-	5.46	3
Co_3O_4	Co	70.0	-	70.0	3
	O	27.1	26.9	27.0	4
	H	0.03	0.04	0.04	0
$Co(OH)_2$	Co	62.5	62.7	62.6	1
	O	34.7	34.9	34.8	2
	H	2.21	2.25	2.23	2
CoOOH	Co	61.3	61.4	61.4	1
	O	36.7	36.6	36.7	2
	H	1.18	1.21	1.20	1

12 Energy-dispersive X-ray spectroscopy (EDX) data

Table S2. EDX data for CoS_x-ox, CoS_x, CoS_x-ox-PhIO, Co_3O_4, $Co(OH)_2$, and CoOOH. All EDX samples were prepared in air for the measurements. Hence, the oxygen content detected by EDX, especially for cobalt sulfide-based samples (CoS_x-ox, CoS_x, CoS_x-ox-PhIO), may have resulted from oxidation in air. Since the Co-based materials were otherwise handled under inert atmosphere in this work, the oxygen content measured by EDX for CoS_x-ox, CoS_x, CoS_x-ox-PhIO should be interpreted with care. EDX measurements also show carbon and sometimes Al signals that probably stem from the carbon tape and Al-holder used to support the samples. The carbon and Al contents account for the missing percentages to give a total of 100 %. The carbon and Al contents have not been included in this table.

Sample	Element	Atomic %	Stoichiometry
CoS_x-ox	Co	37.3	6
	S	30.5	5
	O	6.2	1
CoS_x	Co	34.1	9
	S	20.3	6
	O	3.6	1
CoS_x-ox-PhIO	Co	21.1	2
	S	19.5	2
	O	9.6	1
Co_3O_4	Co	20.4	1
	O	50.7	2.5
$Co(OH)_2$	Co	28.2	1
	O	59.4	2
CoOOH	Co	29.3	1
	O	54.8	2

13 Scanning electron microscopy (SEM) images

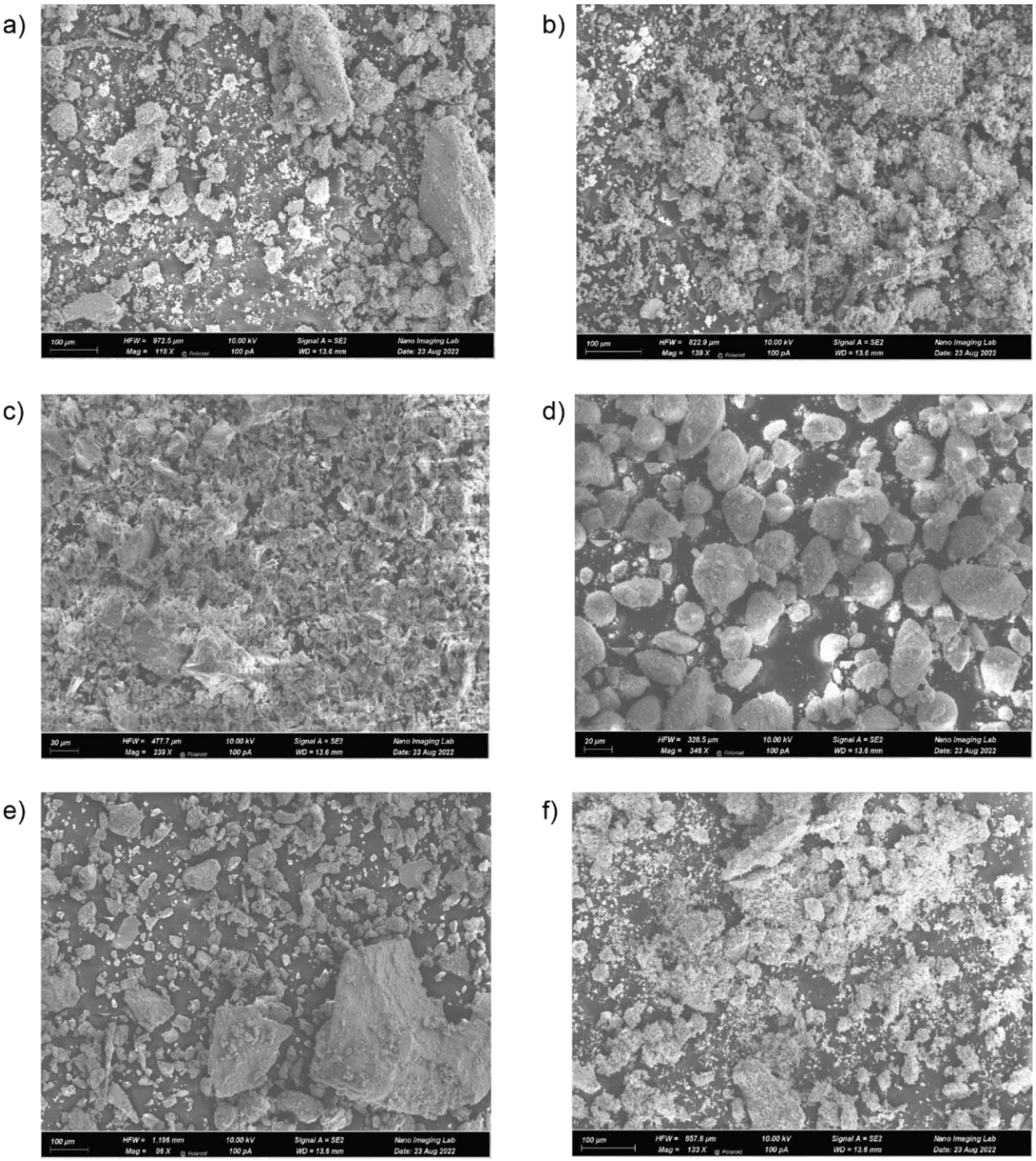

Fig. S14. SEM images of a) CoS_x, b) CoS_x-ox, c) Co_3O_4, d) $Co(OH)_2$, e) CoOOH and f) CoS_x-ox-PhIO. The SEM images show that all examined materials have a large particle size distribution and contain large particles with sizes ranging roughly between few and 500 µm. In the main text we show SEM images of the Co-based materials with larger magnification to highlight surface morphology.

14 N_2 adsorption measurement results

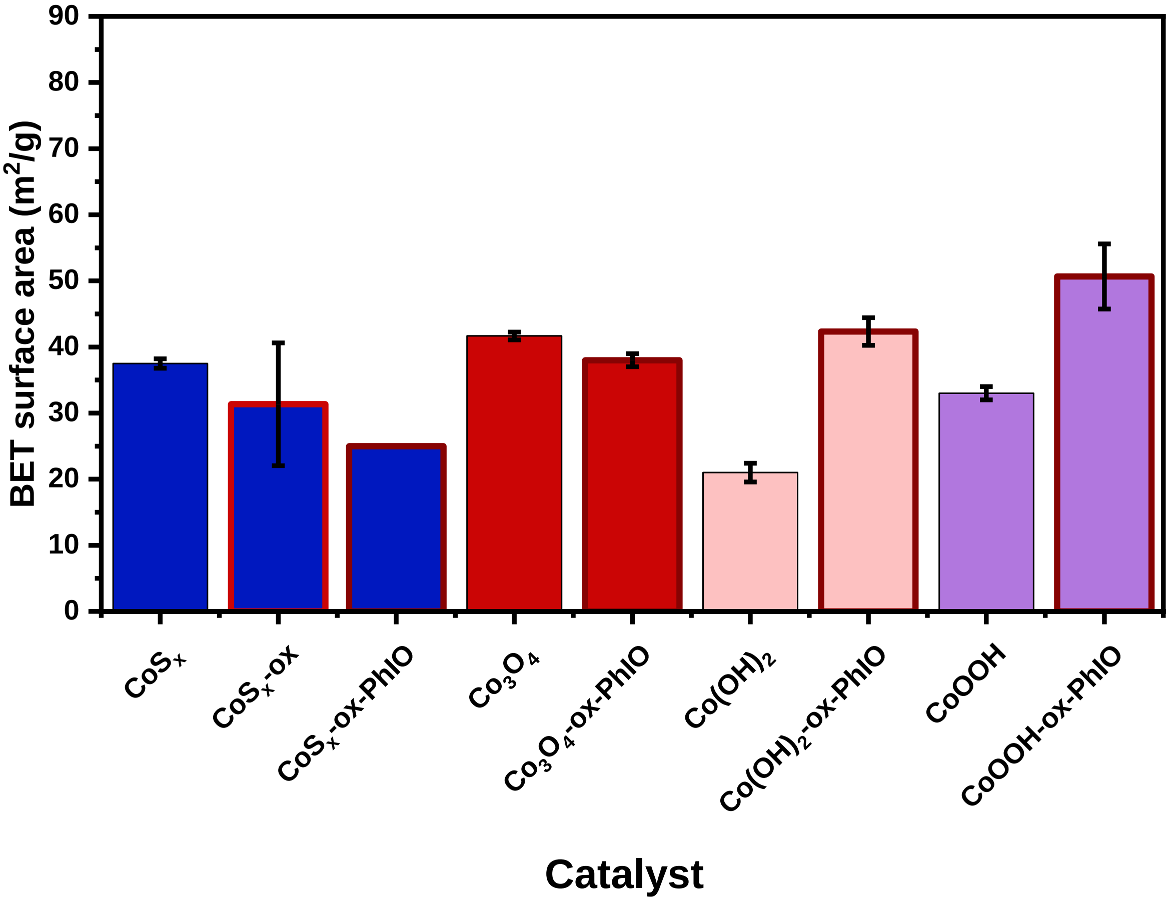

Fig. S15. BET surface areas determined via N_2 adsorption experiments of the as-prepared materials CoS_x, CoS_x-ox, Co_3O_4, $Co(OH)_2$, and CoOOH, and of the Co-based materials after oxidation with 1-(*tert*-butylsulfonyl)2-iodosylbenzene (denoted with the suffix "ox-PhIO", for example "CoS_x-ox-PhIO"). Error bars have been determined from three replicate measurements.

15 Estimated surface roughness

Table S3. This table evaluates the rough radius particles of CoS_x, CoS_x-ox, Co_3O_4, $Co(OH)_2$, and CoOOH would need to have in order explain the measured BET surface area assuming uniform, spherical and smooth particles. We find that the particles would have to be much smaller than what we found by SEM (Fig. S14) with radii on the order of roughly 10-50 nm for the different Co-based materials. *These particle radii are hence inconsistent with our results and suggest that the particles have significant surface roughness* (see Table S4).

**Material**	**CoS_x**	**CoS_x-ox**	**Co_3O_4**	**$Co(OH)_2$**	**CoOOH**
density of material in g/cm^3	5.7	5.7	6.11	3.6	3.79
density of material in g/m^3	5700000	5700000	6110000	3600000	3790000
**expected radius if particles were smooth /m (inconsistent with SEM results)**	**1.40E-08**	**1.70E-08**	**1.20E-08**	**4.00E-08**	**2.40E-08**
surface area per particle in m^2	2.46E-15	3.63E-15	1.81E-15	2.01E-14	7.24E-15
volume per particle in m^3	1.15E-23	2.06E-23	7.24E-24	2.68E-22	5.79E-23
weight per particle in g	6.55E-17	1.17E-16	4.42E-17	9.65E-16	2.19E-16
surface area in m^2/g from particle radius	37.59	30.96	40.92	20.83	32.98
measured surface area in m^2/g	37.5	31.3	41.7	21	33

Table S4. Estimated roughness factor for the surfaces of CoS_x, CoS_x-ox, Co_3O_4, $Co(OH)_2$, and CoOOH assuming the majority of particles have radii of 10 μm or larger (based on SEM data, Fig. S14) and assuming spherical particles. *We find roughness factors on the order of at least 100-1000 are needed to explain the measured surface areas*. Due to the applied assumptions that do not perfectly represent the complex size and shape distribution of particles found experimentally, the derived roughness factors given in the table should only be taken as rough estimates and not as accurate values.

**Material**	**CoS_x**	**CoS_x-ox**	**Co_3O_4**	**$Co(OH)_2$**	**CoOOH**
density of material in g/cm^3	5.45	5.7	6.11	3.6	3.79
density of material in g/m^3	5450000	5700000	6110000	3600000	3790000
particle radius /m	1.00E-05	1.00E-05	1.00E-05	1.00E-05	1.00E-05
surface area per particle in m^2	1.26E-09	1.26E-09	1.26E-09	1.26E-09	1.26E-09
volume per particle in m^3	4.19E-15	4.19E-15	4.19E-15	4.19E-15	4.19E-15
weight per particle in g	2.28E-08	2.39E-08	2.56E-08	1.51E-08	1.59E-08
expected surface area in m^2/g	5.50E-02	5.26E-02	4.91E-02	8.33E-02	7.92E-02
measured surface area in m^2/g	37.5	31.3	41.7	21	33
**roughness factor**	**680**	**600**	**850**	**250**	**420**

16 Estimated fraction of cobalt atoms at the surface

Determining the fraction of cobalt atoms at the surface of the different CoS_x, CoS_x-ox, Co_3O_4, $Co(OH)_2$ and CoOOH is challenging due to the amorphous nature and large size and shape distribution of the corresponding particles. We derive a first, rough estimate of the fraction of cobalt atoms at the surface for the different materials from the measured BET surface area (Fig. S15) and assuming uniform, smooth, and spherical particles with one specific crystal facet mainly exposed. Physical properties and crystallographic data were retrieved from the Inorganic Crystal Structure Database (ICSD). This is shown for CoS_x in Fig. S16 and Table S5, and similar considerations were applied for the other Co-based materials.

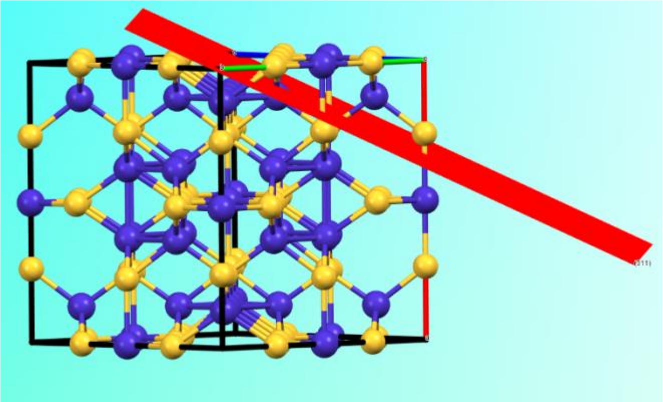

Fig. S16. Crystal structure of the unit cell of Co_9S_8. The computed crystal plane (311) (red) cuts through one single cobalt atom. Computations were conducted using the program Mercury.

Table S5. Estimated fraction of the cobalt atoms exposed at the surface of CoS_x.

	**Source/Formula**	**CoS_x**
Measured surface area / m^2 per g	BET measurement	37.5
Density of CoS_x (assuming a Co_9S_8 composition) g per cm^3, rho	ICSD	5.7
Side length (unit cell) Å	ICSD	9.9
Area of unit cell facet (311) / Å^2	ICSD	34.6
N(Co) on the unit cell facet (311)	ICSD	1
Volume (unit cell) / Å^3	ICSD	977
N (Co) per unit cell	ICSD	35
Particle radius / m	3/(rho*10^6*A(BET))	1.4*10^{-8}
Particle radius r / Å		140
Surface area per particle / Å^2	4*π*r^2	247537
N(surface Co) per particle	A(Particle)/A((311))	7160
Volume of particle / Å^3	4/3 *π*r^3	11580678
N(Co) per particle	V(Particle)/(V(unitcell)/#Co)	414832
**Estimated fraction of surface Co / %**	**N(surfCo)/N(Co)*100**	**1.7**

17 X-ray photoelectron spectroscopy (XPS) data

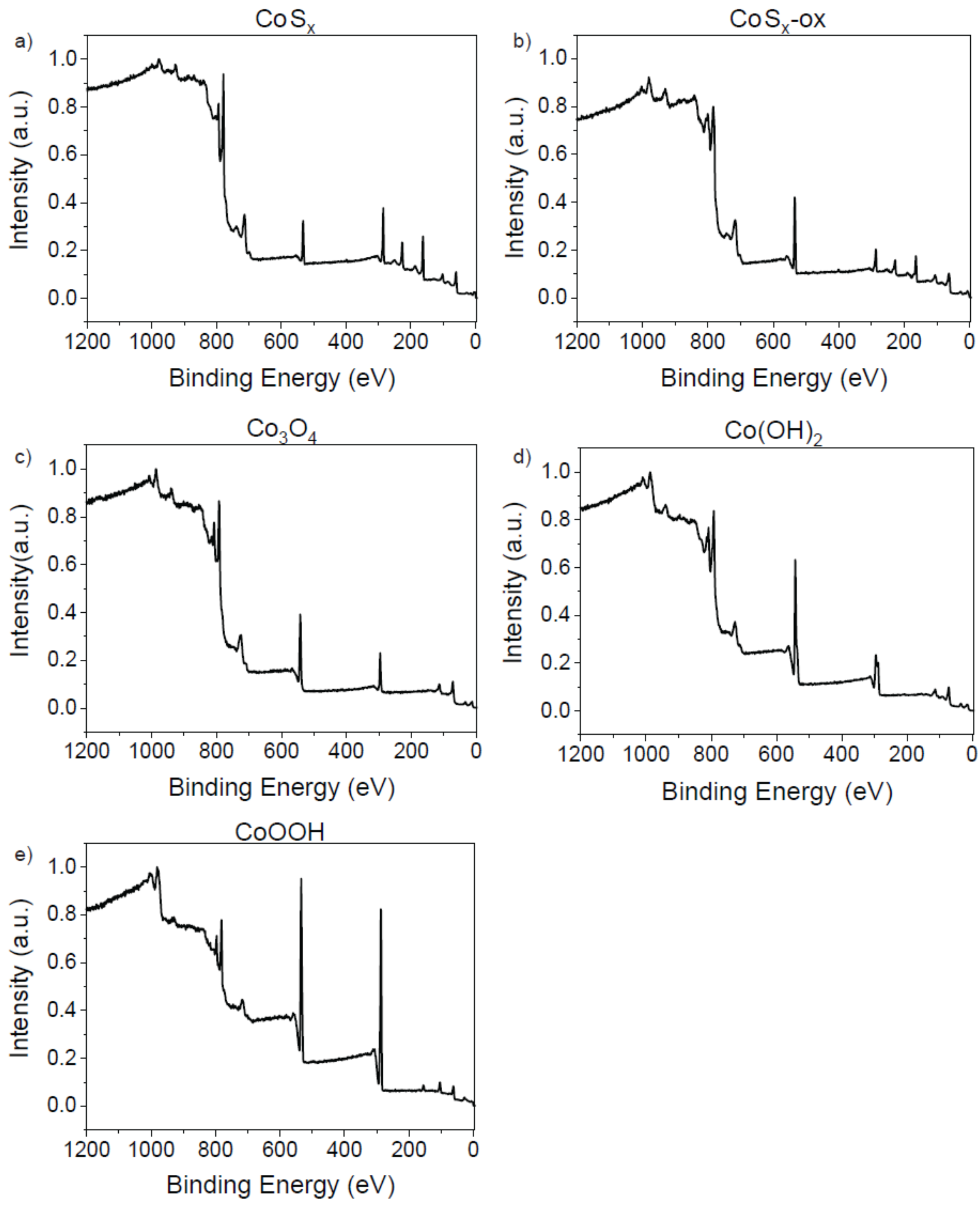

Fig. S17. XPS survey spectra of a) CoS_x, b) CoS_x-ox, c) Co_3O_4, d) $Co(OH)_2$, and e) CoOOH. High-resolution scans are shown in the main text.

Discussion of the contribution of carbon tape to XPS spectra.

Carbon tape was used as a support for XPS analysis of the different materials. XPS analysis of this carbon tape showed that we should expect contributions to both C1s and O1s spectra from the carbon tape with a C/O ratio of roughly 1:0.4 (Table S6 and Fig. S18). Therefore, any O1s signal observed in the analysis of our materials will be convoluted with oxygen present on the carbon paper support. By determining the ratio of carbon to oxygen on the surface of our materials, we tried to account for the contribution of carbon paper to the observed O1s signal for the different materials. For example, XPS analysis of CoS_x suggested a C:O ratio of ~1:0.9. Hence, to a first approximation, up to roughly half of the observed O1s signal may be due to the carbon tape support and only roughly half may be due to oxygen on the surface of CoS_x. However, such considerations can only give a rough idea of the contribution of the carbon tape to the O1s signals measured for the Co-based materials, because the oxygen content on carbon paper can vary even for the same type of carbon paper, and because the XPS data may also be influenced by the specific amount and distribution of deposited sample on the carbon support. Therefore, O1s data of all materials examined herein should be interpreted with care.

The fits shown in the main text of the O1s XPS spectra include a signal from carbon tape at ~531.6 eV, where the O1s maximum was found for the bare carbon tape (Fig. S18), and thereby account for the contribution from the carbon tape.

Table S6. Ratio of the integrated C1s and O1s XPS signals for carbon paper, CoS_x, CoS_x-ox, Co_3O_4, $Co(OH)_2$, and CoOOH. Note that XPS data of CoS_x-ox was measured using a different type of carbon paper support that was stored under air and that may contain a higher surface oxygen content.

Ratio	C1s peak area	O1s peak area
Carbon paper	1	0.4
CoS_x	1	0.9
CoS_x-ox	1	3.4
Co_3O_4	1	2
$Co(OH)_2$	1	1.8
CoOOH	1	1.1

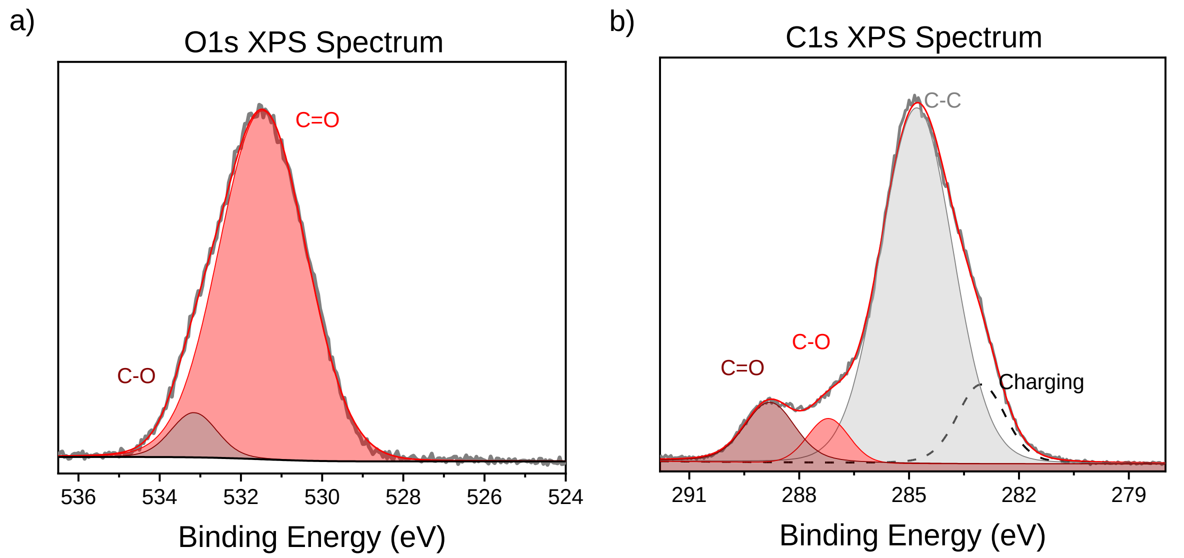

Fig. S18. XPS O1s and C1s spectra of carbon tape used as a support for XPS analysis.

18 Infrared (IR) spectroscopy data

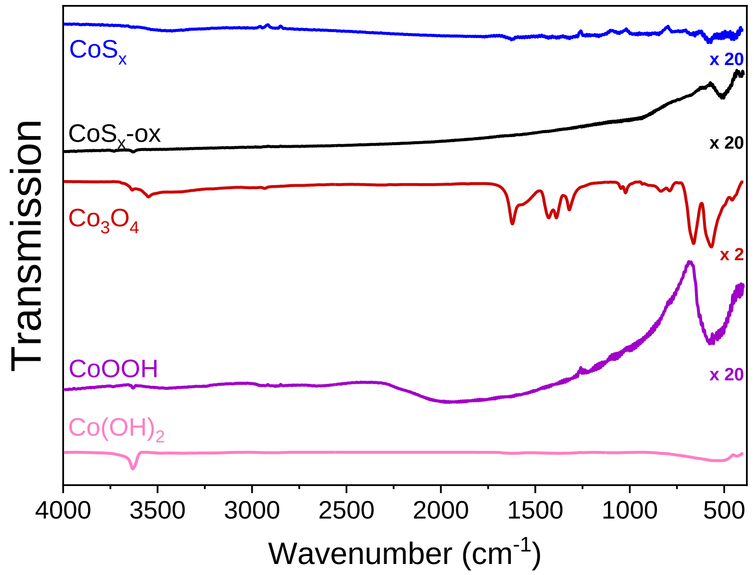

Fig. S19. IR spectra of CoS_x (blue), CoS_x-ox (black), Co_3O_4 (red), CoOOH (purple), and $Co(OH)_2$ (pink) showing the full wavenumber range measured from 4000-400 cm^{-1}. Zooms in the region of 3000-4000 cm^{-1} are shown in the main text in Fig. 2d. Individual IR spectra have been vertically scaled as indicated on the right.

19 NMR spectroscopy data from the epoxidation of cyclooctene by PhIO

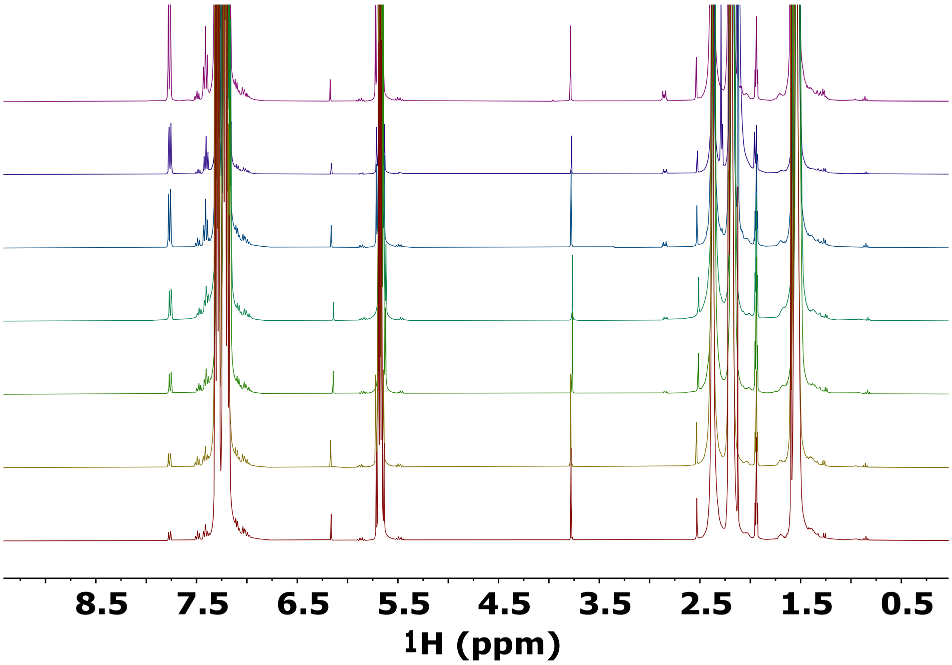

Fig. S20. ^{1}H-NMR spectra (400 MHz, MeCN-*d*$_3$) of the reaction solution from a representative epoxidation of cyclooctene to cyclooctene oxide by PhIO using CoS_x-ox. Samples were taken at t = 0, t = 5 min, t = 15 min, t = 30 min, t = 1 h, t = 3 h and t = 5 h (from bottom to top). The signal at 2.84 ppm was used to quantify the amount of cyclooctene oxide formed and corresponds to the two protons of the oxirane group. The signal at ~7.7 ppm was used to quantify the amount of PhI, corresponding the two aromatic protons at 2 and 6 position. The signal at 7.4 ppm corresponds to one aromatic proton at the 4 position.

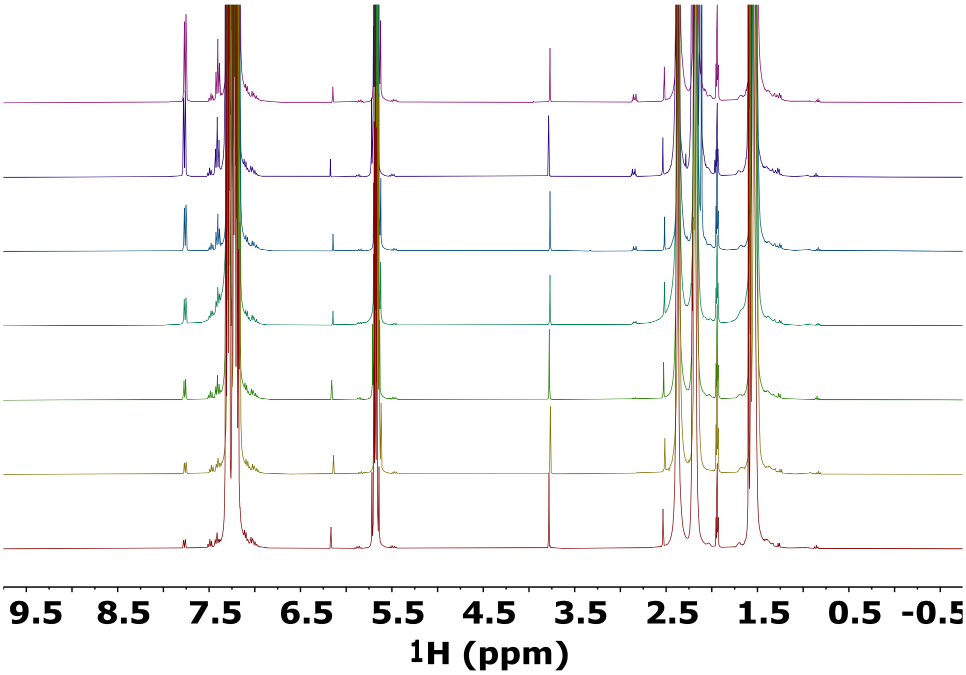

Fig. S21. ^{1}H-NMR spectra (400 MHz, MeCN-*d*$_3$) of the reaction solution from a representative epoxidation of cyclooctene to cyclooctene oxide by PhIO using CoS_x. Samples were taken at t = 0, t = 5 min, t = 15 min, t = 30 min, t = 1 h, t = 3 h and t = 5 h (from bottom to top). The signal at 2.84 ppm was used to quantify the amount of cyclooctene oxide formed and corresponds to the two protons of the oxirane group. The signal at ~7.7 ppm was used to quantify the amount of PhI, corresponding the two aromatic protons at 2 and 6 position. The signal at 7.4 ppm corresponds to one aromatic proton at the 4 position.

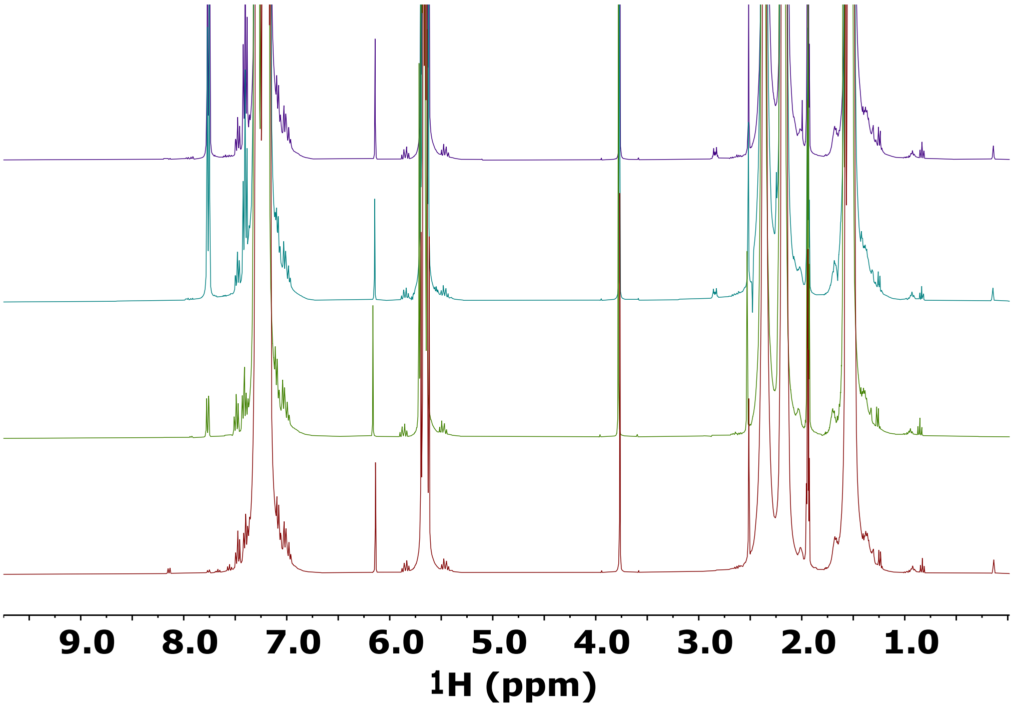

Fig. S22. ^{1}H-NMR spectra (400 MHz, MeCN-d_3) of the reaction solution from a representative epoxidation of cyclooctene to cyclooctene oxide by PhIO using Co_3O_4. Samples were taken at t = 0, t = 5 min, t = 1 h and t = 5 h (from bottom to top). The signal at 2.84 ppm was used to quantify the amount of cyclooctene oxide formed and corresponds to the two protons of the oxirane group. The signal at ~7.7 ppm was used to quantify the amount of PhI, corresponding the two aromatic protons at 2 and 6 position. The signal at 7.4 ppm corresponds to one aromatic proton at the 4 position.

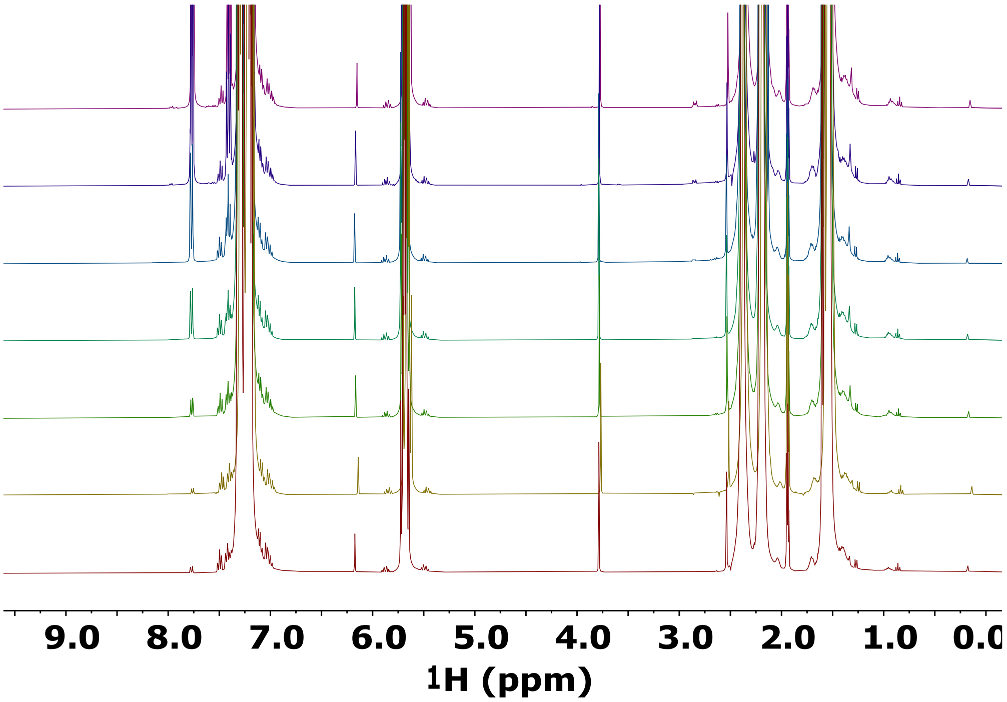

Fig. S23. ^{1}H-NMR spectra (400 MHz, MeCN-d_3) of the reaction solution from a representative epoxidation of cyclooctene to cyclooctene oxide by PhIO using CoOOH. Samples were taken at t = 0, t = 5 min, t = 15 min, t = 30 min, t = 1 h, t = 3 h and t = 5 h (from bottom to top). The signal at 2.84 ppm was used to quantify the amount of cyclooctene oxide formed and corresponds to the two protons of the oxirane group. The signal at ~7.7 ppm was used to quantify the amount of PhI, corresponding the two aromatic protons at 2 and 6 position. The signal at 7.4 ppm corresponds to one aromatic proton at the 4 position.

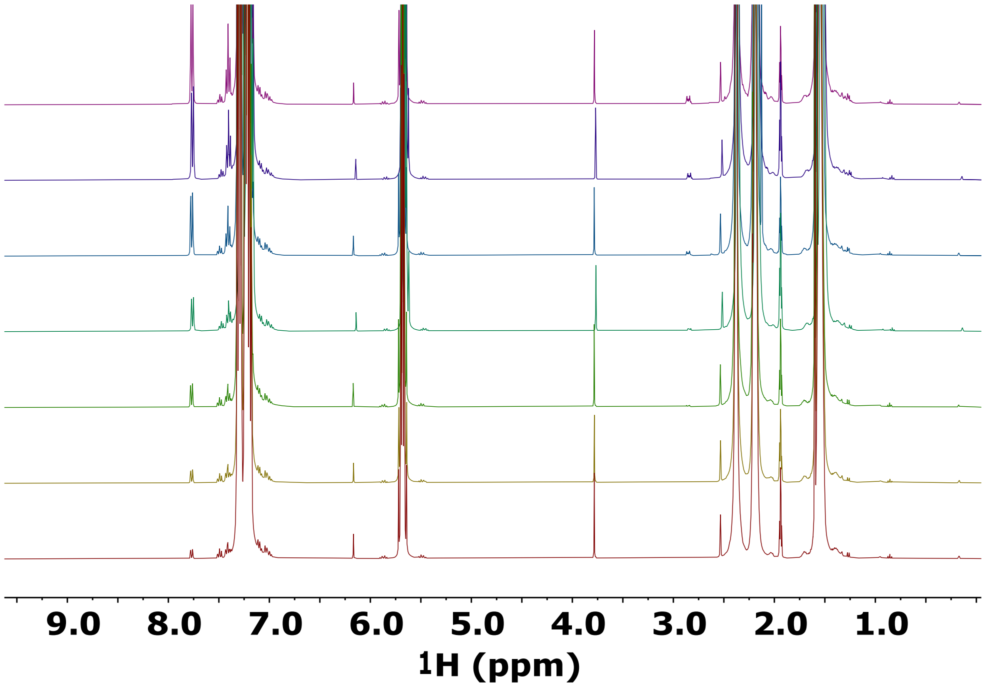

Fig. S24. ^{1}H-NMR spectra (400 MHz, MeCN-d_3) of the reaction solution from a representative epoxidation of cyclooctene to cyclooctene oxide by PhIO using $Co(OH)_2$. Samples were taken at t = 0, t = 5 min, t = 15 min, t = 30min, t = 1 h, t = 3 h and t = 5 h (from bottom to top). The signal at 2.84 ppm was used to quantify the amount of cyclooctene oxide formed and corresponds to the two protons of the oxirane group. The signal at ~7.7 ppm was used to quantify the amount of PhI, corresponding the two aromatic protons at 2 and 6 position. The signal at 7.4 ppm corresponds to one aromatic proton at the 4 position.

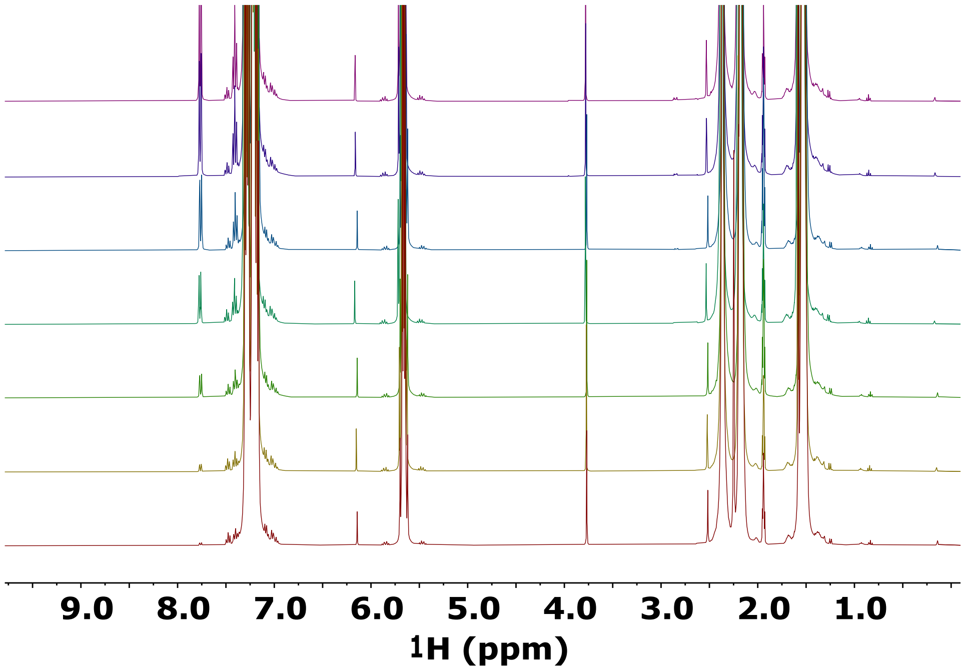

Fig. S25. ^{1}H-NMR (400 MHz, MeCN-d_3) spectra of the reaction solution from a representative epoxidation of cyclooctene to cyclooctene oxide by PhIO in the absence of a catalyst. Samples were taken at t = 0, t = 5 min, t = 15 min, t = 30 min, t = 1 h, t = 3 h and t = 5 h (from bottom to top). The signal at 2.84 ppm was used to quantify the amount of cyclooctene oxide formed and corresponds to the two protons of the oxirane group. The signal at ~7.7 ppm was used to quantify the amount of PhI, corresponding the two aromatic protons at 2 and 6 position. The signal at 7.4 ppm corresponds to one aromatic proton at the 4 position.

20 NMR spectroscopy data from the epoxidation of cyclooctene by ^{t}BuOOH

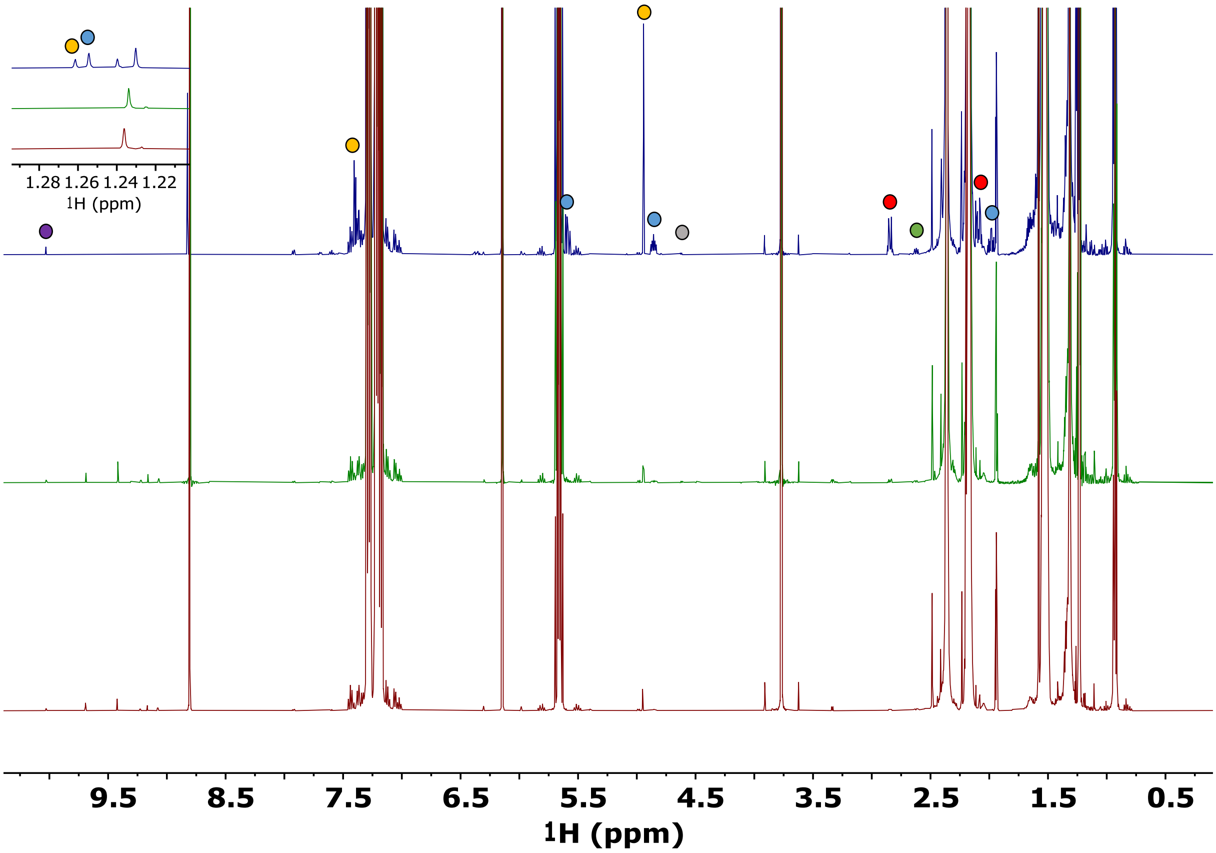

Fig. S26. ^{1}H-NMR (500 MHz, MeCN-d_3) spectra of the reaction solution from a representative epoxidation of cyclooctene to cyclooctene oxide by ^{t}BuOOH using CoS_x. Samples were taken at t = 0, t = 5 min and t = 5 h (from bottom to top). NMR signals marked with colored dots have been used to quantify the different products formed. The signal at 2.87 ppm was used to quantify the amount of cyclooctene oxide (red dot) formed and corresponds to the two protons of the oxirane group. The signal at 2.62 ppm was used to quantify the amount of cyclooct-2-en-1-one (green dot) and corresponds to two methylene protons in alpha position to the C=O group. The singlet signals at 1.25 and 1.26 ppm correspond to *tert*-butyl groups of 3-(*tert*-butylperoxy)cyclooct-1-ene (blue dot) and ((*tert*-butylperoxy)methyl)benzene (yellow dot), respectively, and were used for their quantification. The singlet signal at 10 ppm was used to quantify the amount of benzaldehyde (purple dot) and the signal at 4.6 ppm was used to quantify the amount of benzyl alcohol (grey dot). Due to the complexity of the obtained NMR spectra we also used GC-MS to assist the identification of the formed products.

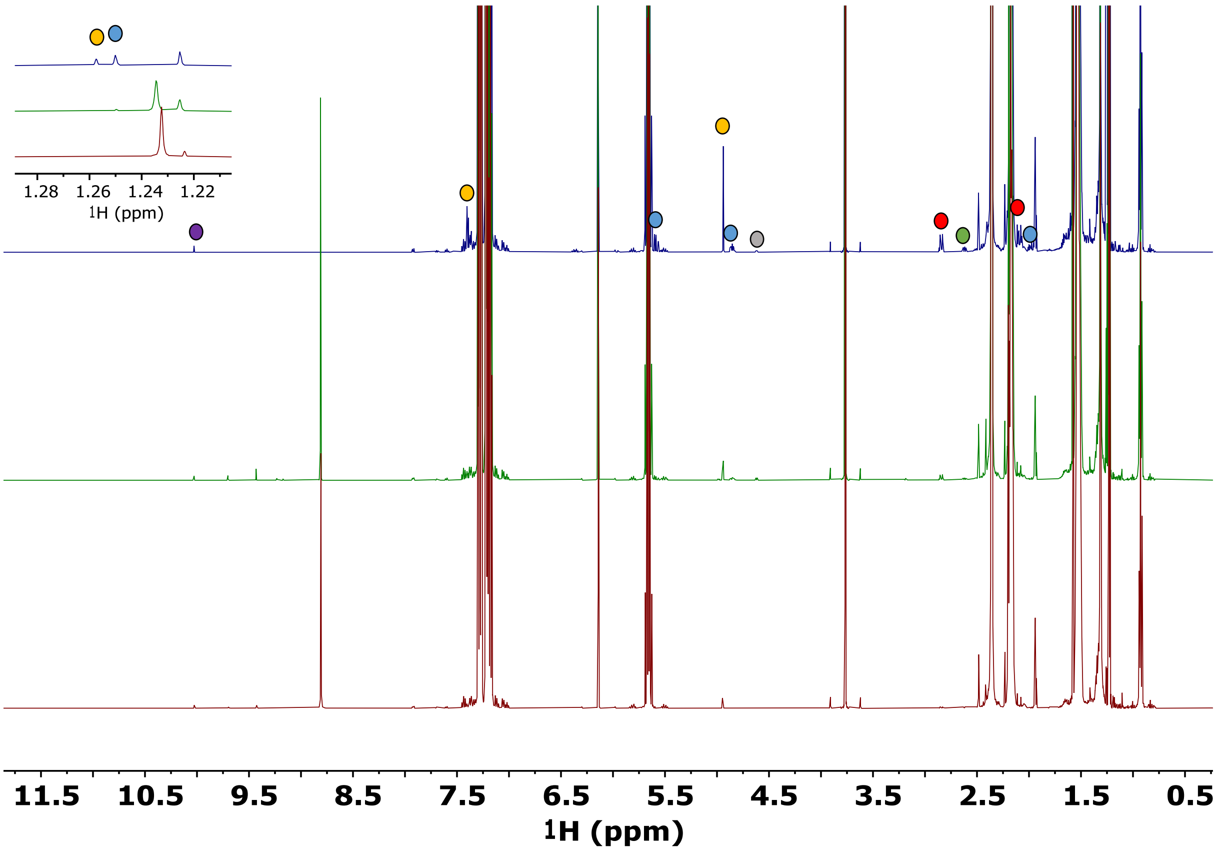

Fig. S27. ^{1}H-NMR (500 MHz, MeCN-*d*$_3$) spectra of the reaction solution from a representative epoxidation of cyclooctene to cyclooctene oxide by ^{t}BuOOH using CoS_x-ox. Samples were taken at t = 0, t = 5 min and t = 5 h (from bottom to top). NMR signals marked with colored dots have been used to quantify the different products formed. The signal at 2.87 ppm was used to quantify the amount of cyclooctene oxide (red dot) formed and corresponds to the two protons of the oxirane group. The signal at 2.62 ppm was used to quantify the amount of cyclooct-2-en-1-one (green dot) and corresponds to two methylene protons in alpha position to the C=O group. The singlet signals at 1.25 and 1.26 ppm correspond to *tert*-butyl groups of 3-(*tert*-butylperoxy)cyclooct-1-ene (blue dot) and ((*tert*-butylperoxy)methyl)benzene (yellow dot), respectively, and were used for their quantification. The singlet signal at 10 ppm was used to quantify the amount of benzaldehyde (purple dot) and the signal at 4.6 ppm was used to quantify the amount of benzyl alcohol (grey dot). Due to the complexity of the obtained NMR spectra we also used GC-MS to assist the identification of the formed products.

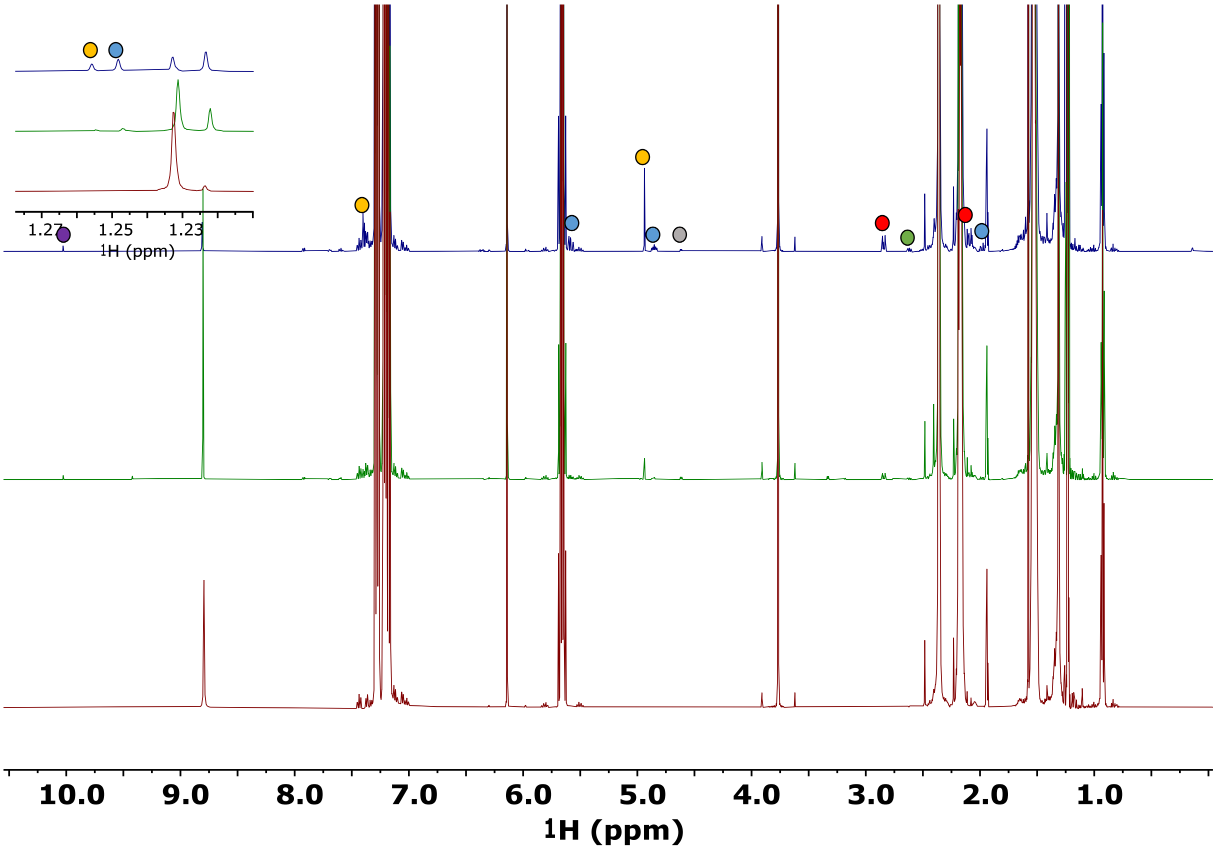

Fig. S28. ^{1}H-NMR (500 MHz, MeCN-d_3) spectra of the reaction solution from a representative epoxidation of cyclooctene to cyclooctene oxide by ^{t}BuOOH using Co_3O_4. Samples were taken at t = 0, t = 5 min and t = 5 h (from bottom to top). NMR signals marked with colored dots have been used to quantify the different products formed. The signal at 2.87 ppm was used to quantify the amount of cyclooctene oxide (red dot) formed and corresponds to the two protons of the oxirane group. The signal at 2.62 ppm was used to quantify the amount of cyclooct-2-en-1-one (green dot) and corresponds to two methylene protons in alpha position to the C=O group. The singlet signals at 1.25 and 1.26 ppm correspond to *tert*-butyl groups of 3-(*tert*-butylperoxy)cyclooct-1-ene (blue dot) and ((*tert*-butylperoxy)methyl)benzene (yellow dot), respectively, and were used for their quantification. The singlet signal at 10 ppm was used to quantify the amount of benzaldehyde (purple dot) and the signal at 4.6 ppm was used to quantify the amount of benzyl alcohol (grey dot). Due to the complexity of the obtained NMR spectra we also used GC-MS to assist the identification of the formed products.

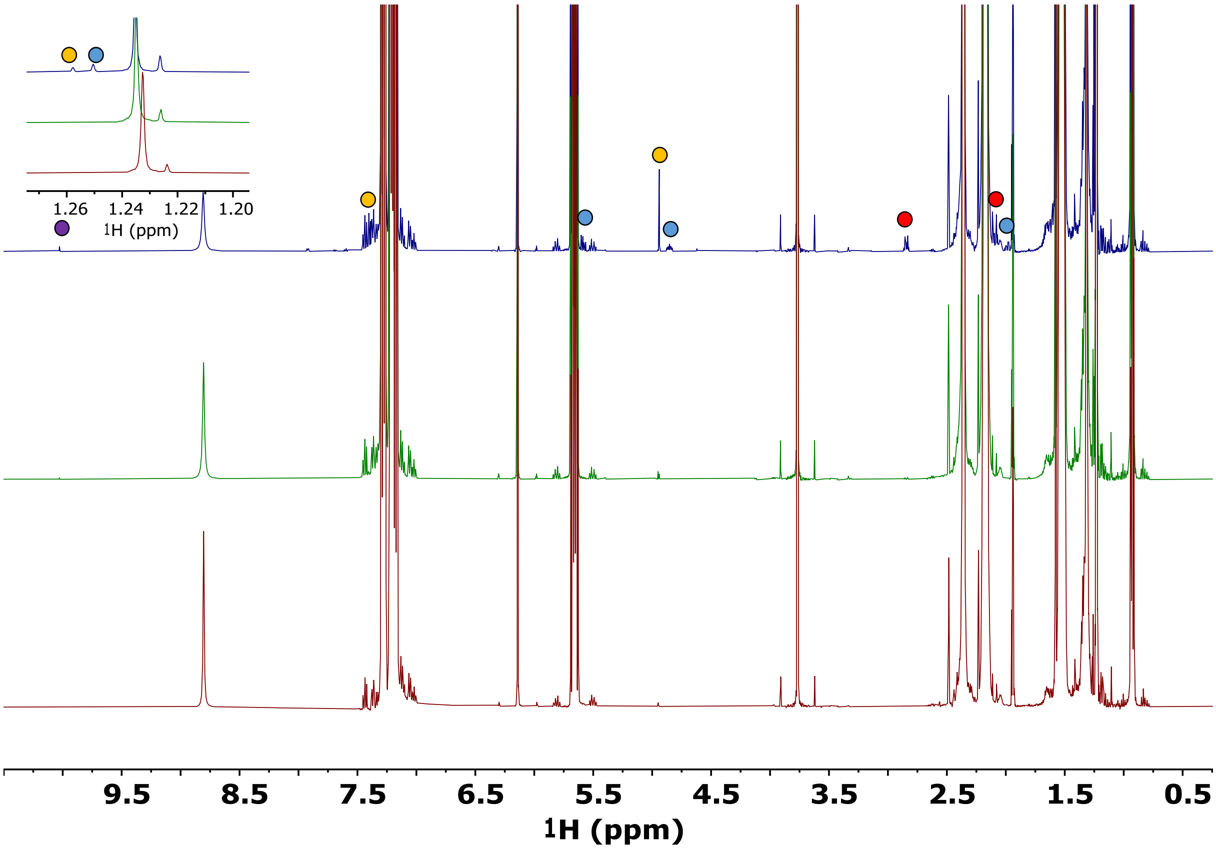

Fig. S29. ^{1}H-NMR (500 MHz, MeCN-*d*$_3$) spectra of the reaction solution from a representative epoxidation of cyclooctene to cyclooctene oxide by ^{t}BuOOH using CoOOH. Samples were taken at t = 0, t = 5 min and t = 5 h (from bottom to top). NMR signals marked with colored dots have been used to quantify the different products formed. The signal at 2.87 ppm was used to quantify the amount of cyclooctene oxide (red dot) formed and corresponds to the two protons of the oxirane group. The singlet signals at 1.25 and 1.26 ppm correspond to *tert*-butyl groups of 3-(*tert*-butylperoxy)cyclooct-1-ene (blue dot) and ((*tert*-butylperoxy)methyl)benzene (yellow dot), respectively, and were used for their quantification. The singlet signal at 10 ppm was used to quantify the amount of benzaldehyde (purple dot). Due to the complexity of the obtained NMR spectra we also used GC-MS to assist the identification of the formed products.

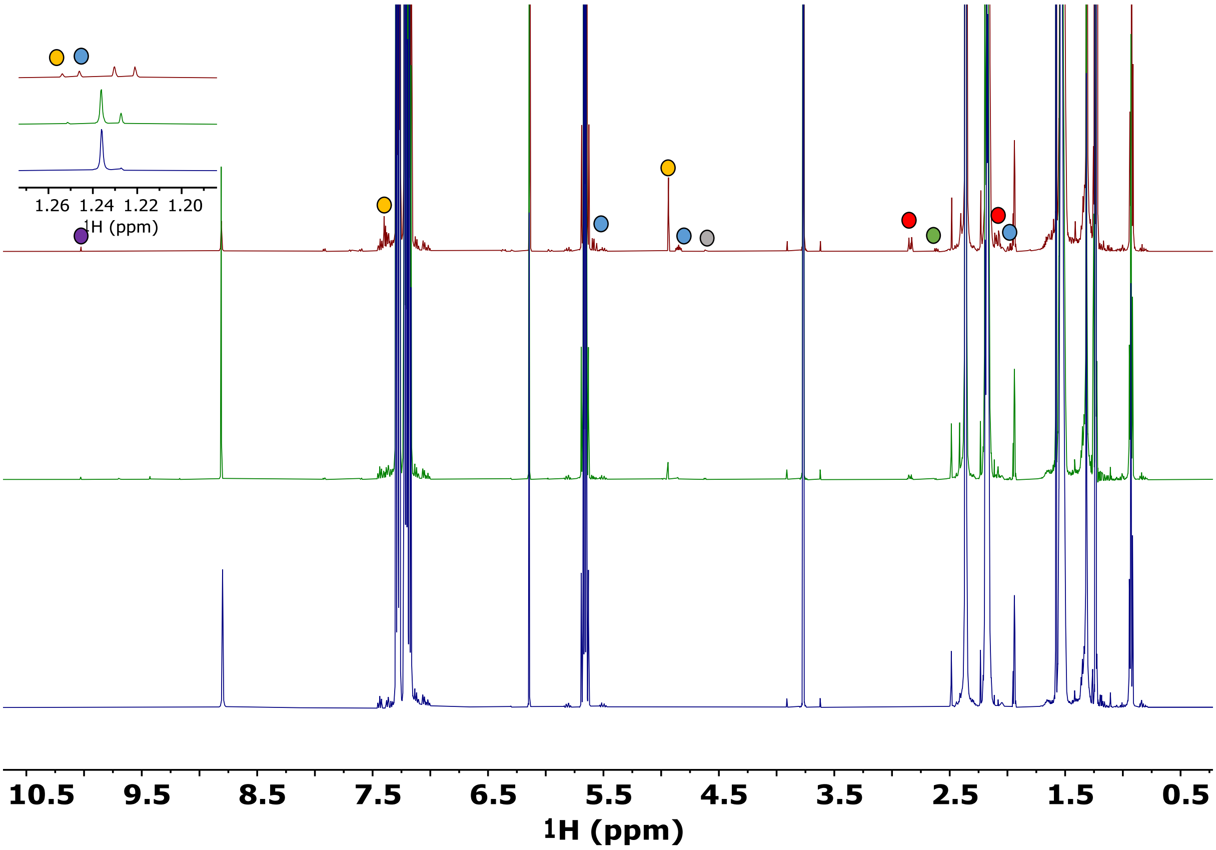

Fig. S30. ^{1}H-NMR (500 MHz, MeCN-*d*$_3$) spectra of the reaction solution from a representative epoxidation of cyclooctene to cyclooctene oxide by ^{t}BuOOH using $Co(OH)_2$. Samples were taken at t = 0, t = 5 min and t = 5 h (from bottom to top). NMR signals marked with colored dots have been used to quantify the different products formed. The signal at 2.87 ppm was used to quantify the amount of cyclooctene oxide (red dot) formed and corresponds to the two protons of the oxirane group. The signal at 2.62 ppm was used to quantify the amount of cyclooct-2-en-1-one (green dot) and corresponds to two methylene protons in alpha position to the C=O group. The singlet signals at 1.25 and 1.26 ppm correspond to *tert*-butyl groups of 3-(*tert*-butylperoxy)cyclooct-1-ene (blue dot) and ((*tert*-butylperoxy)methyl)benzene (yellow dot), respectively, and were used for their quantification. The singlet signal at 10 ppm was used to quantify the amount of benzaldehyde (purple dot) and the signal at 4.6 ppm was used to quantify the amount of benzyl alcohol (grey dot). Due to the complexity of the obtained NMR spectra we also used GC-MS to assist the identification of the formed products.

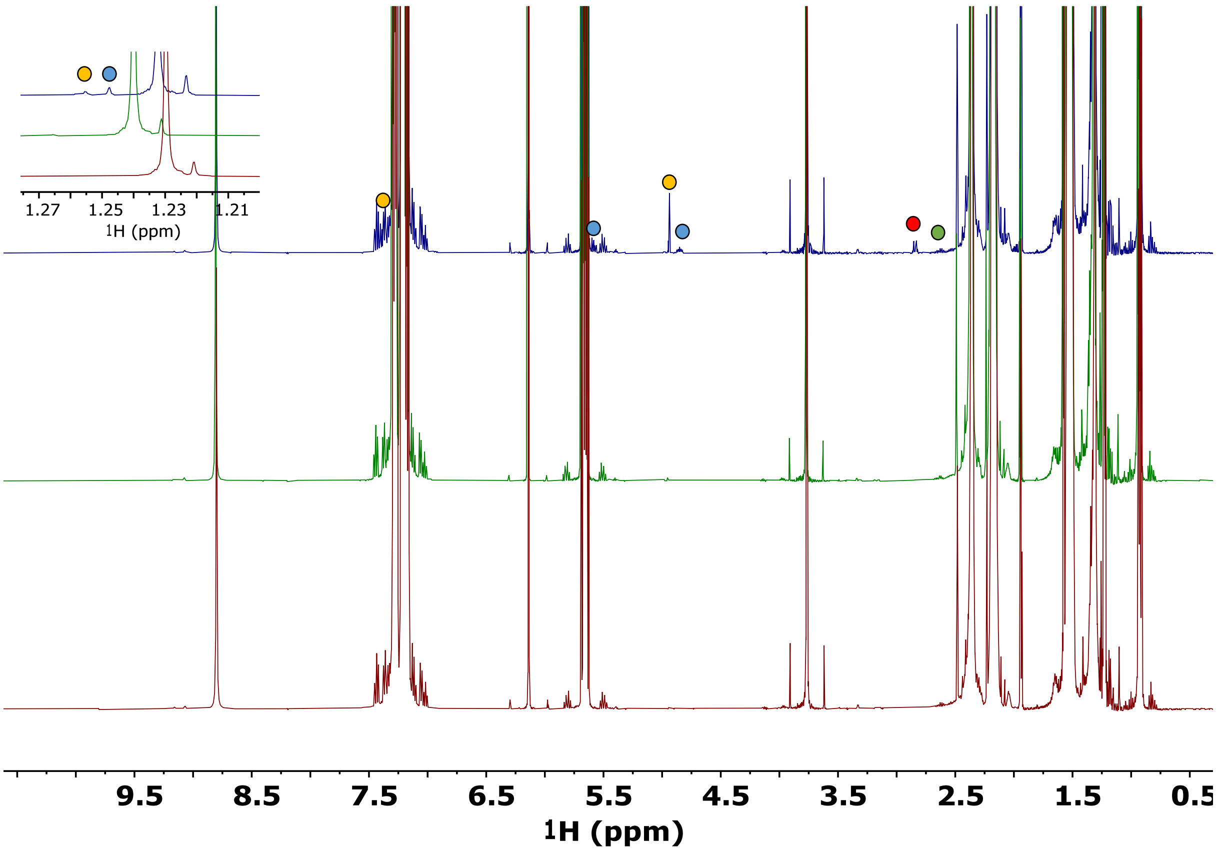

Fig. S31. ^{1}H-NMR (500 MHz, MeCN-*d*$_3$) spectra of the reaction solution from a representative epoxidation of cyclooctene to cyclooctene oxide by ^{t}BuOOH in absence of a material. Samples were taken at t = 0, t = 5 min and t = 5 h (from bottom to top). NMR signals marked with colored dots have been used to quantify the different products formed. The signal at 2.87 ppm was used to quantify the amount of cyclooctene oxide (red dot) formed and corresponds to the two protons of the oxirane group. The signal at 2.62 ppm was used to quantify the amount of cyclooct-2-en-1-one (green dot) and corresponds to two methylene protons in alpha position to the C=O group. The singlet signals at 1.25 and 1.26 ppm correspond to *tert*-butyl groups of 3-(*tert*-butylperoxy)cyclooct-1-ene (blue dot) and ((*tert*-butylperoxy)methyl)benzene (yellow dot), respectively, and were used for their quantification. Due to the complexity of the obtained NMR spectra we also used GC-MS to assist the identification of the formed products.

21 NMR spectroscopy data from the epoxidation of *trans*-2-octene by PhIO

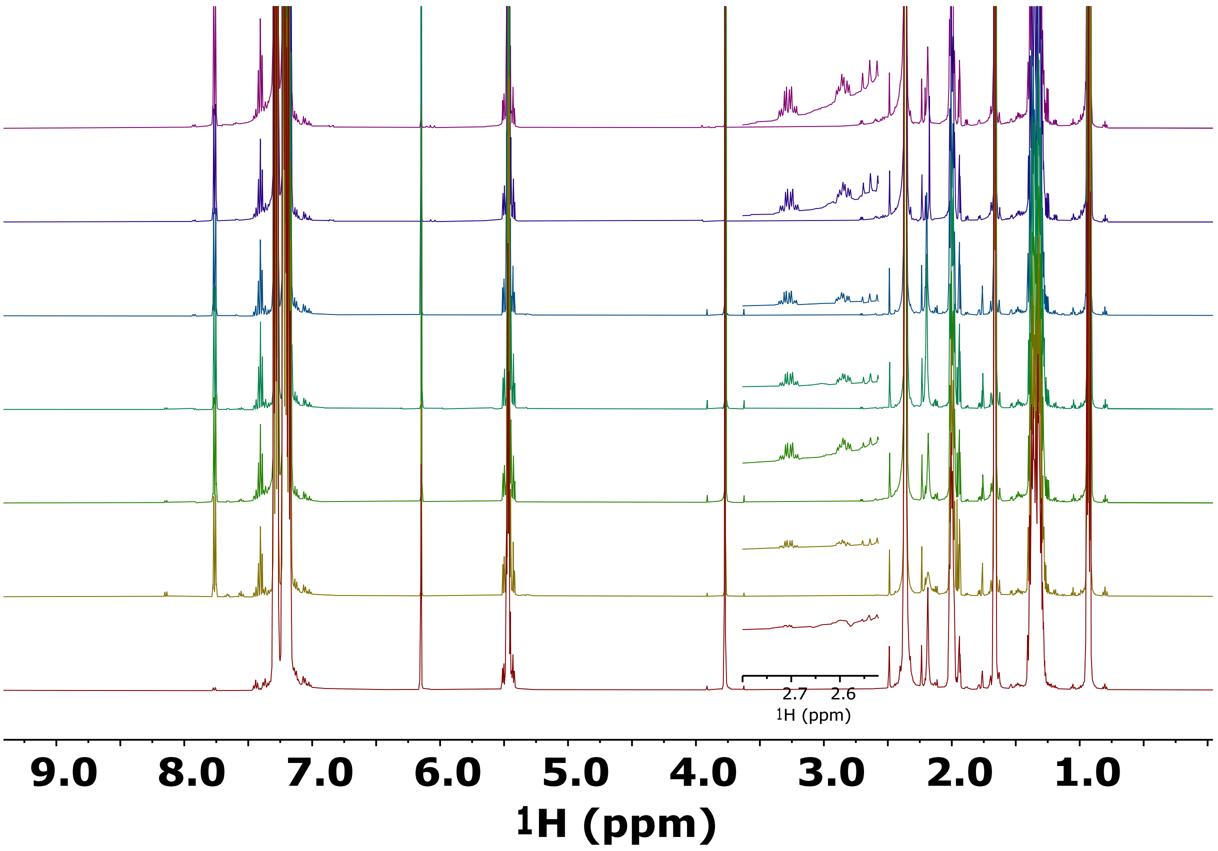

Fig. S32. ^{1}H-NMR (500 MHz, MeCN-d_3) spectra of the reaction solution from a representative epoxidation of *trans*-2-octene by CoS_x, CoS_x-ox, Co_3O_4, CoOOH, $Co(OH)_2$, and a control in the absence of a catalyst after 5 h reaction time (from top to second from the bottom). The red spectrum on the very bottom provides a representative spectrum at the beginning of the epoxidation catalysis, here using $Co(OH)_2$ (with t = 1 min). The quartet-doublet at 2.7 ppm was used to quantify the amount of *E*-2-methyl-3-pentyloxirane formed and corresponds to the one proton of the oxirane group at the 2 position. The triplet-doublet at 2.6 ppm corresponds to the one proton of the oxirane group at the 3 position. A baseline correction was conducted prior to integration. The signal at ~7.7 ppm was used to quantify the amount of PhI, corresponding the two aromatic protons at 2 and 6 position. The signal at 7.4 ppm corresponds to one aromatic proton at the 4 position.

22 NMR spectroscopy data from the epoxidation of *cis*-2-octene by PhIO

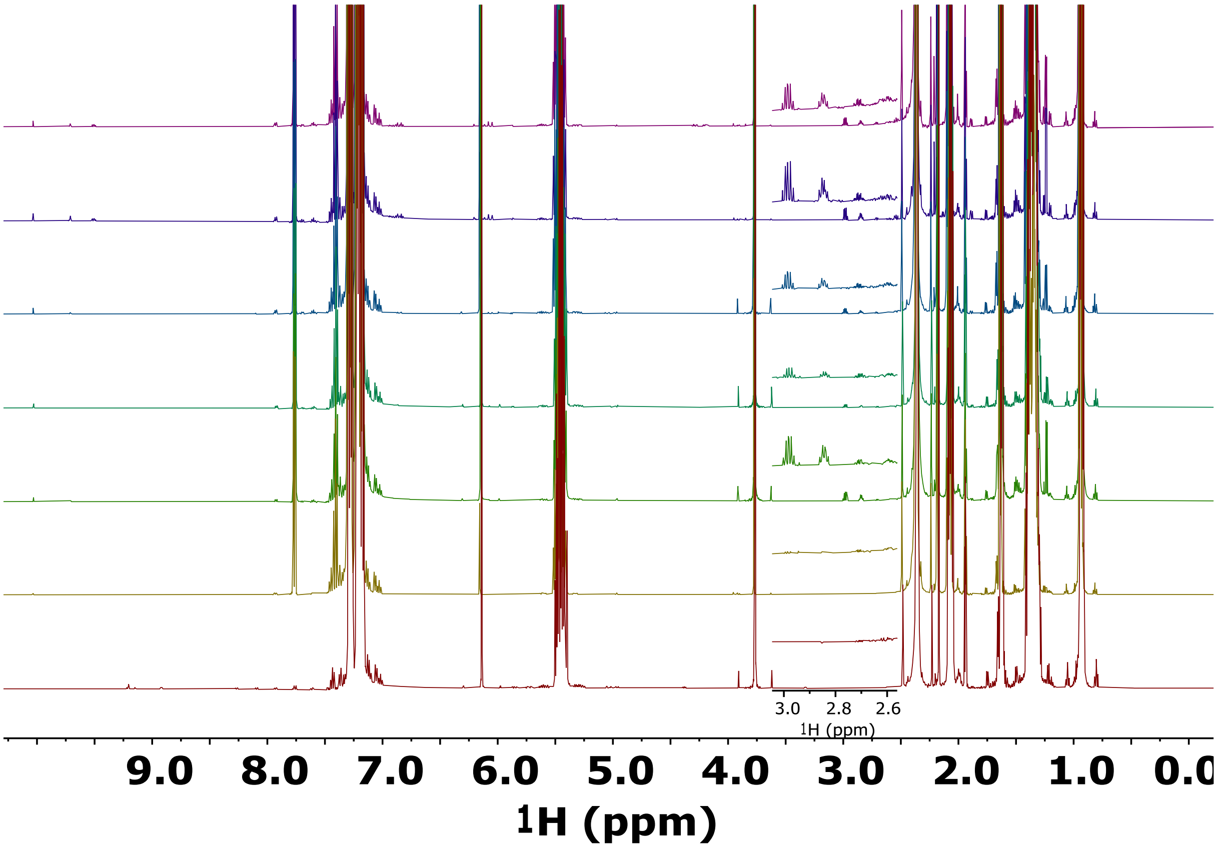

Fig. S33. ^{1}H-NMR (500 MHz, MeCN-d_3) spectra of the reaction solution from a representative epoxidation of *cis*-2-octene by CoS_x, CoS_x-ox, Co_3O_4, CoOOH, $Co(OH)_2$, and a control in the absence of a catalyst after 5 h reaction time (from top to the second from the bottom). The red spectrum on the very bottom provides a representative spectrum at the beginning of the epoxidation catalysis, here using $Co(OH)_2$ (with t = 1 min). The signal at 3.0 ppm was used to quantify the amount of *Z*-2-methyl-3-pentyloxirane formed and corresponds to the one proton of the oxirane group at the 2 position. The signal at 2.84 ppm corresponds to the one proton of the oxirane group at the 3 position. The doublet signal at 1.23 ppm corresponds to the methyl group at the 1 position. The signal at 2.7 ppm was used to quantify the amount of *E*-2-methyl-3-pentyloxirane formed and corresponds to the one proton of the oxirane group at the 2 position. A baseline correction was conducted prior to integration. The signal at ~7.7 ppm was used to quantify the amount of PhI, corresponding the two aromatic protons at 2 and 6 position. The signal at 7.4 ppm corresponds to one aromatic proton at the 4 position.

23 Electron paramagnetic resonance (EPR) spectroscopy data

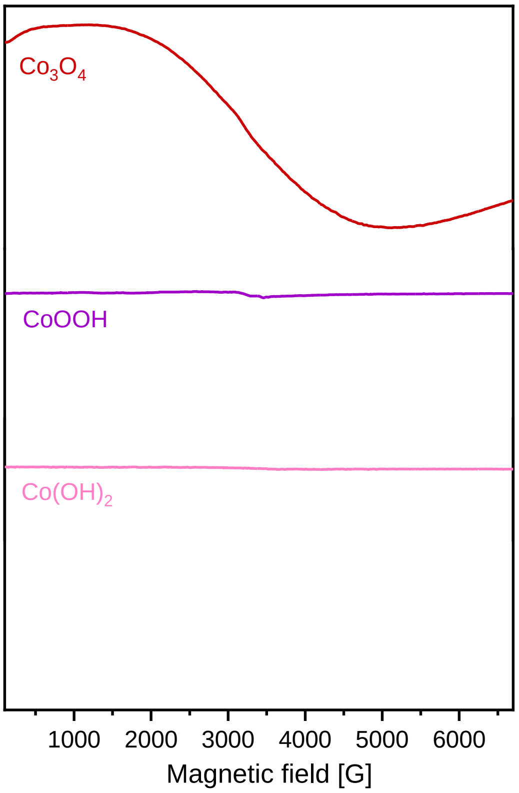

Fig. S34. EPR spectra of as-prepared Co_3O_4 (red), CoOOH (purple), and $Co(OH)_2$ (pink). In the main text (Fig. 1c) we show the same data, but with the EPR spectra of as-prepared CoOOH and $Co(OH)_2$ vertically scaled to show minor signals.

24 Plausible mechanism of the epoxidation of cyclooctene by ^{t}BuOOH

The observation of 3-*tert*-butylperoxycyclooct-1-ene as a major product under epoxidation conditions using ^{t}BuOOH with all Co-based materials (see Fig. S9) suggests a radical chain mechanism involving peroxy radicals generated from a Haber-Weiss type decomposition at Co(II) sites of the catalytic material (Scheme S1), as has previously been proposed for other Co-based catalysts.[2,4-6] The Haber-Weiss mechanism likely leads to the intermediate formation of Co–OO^{t}Bu species at the catalyst surface.[2,4-6] It has previously been suggested that either the O–O or the Co–O bond can be cleaved in the Co–OO^{t}Bu, where the latter is thought more likely in presence of a rigid coordination chemistry and steric shielding at the Co center.[4]

The ^{t}BuOO· and ^{t}BuO· radicals formed via the Haber-Weiss mechanism likely lead predominantly to the observed allylic oxidation products (and the benzylic oxidation products from the solvent toluene), and also to the formation of cyclooctene oxide (Scheme S2).[4] Substrate oxidation by the freely diffusing peroxy and oxy radicals is consistent with the observed inhibition of (ep)oxidation catalysis in presence of a radical scavenger (Fig. S10).

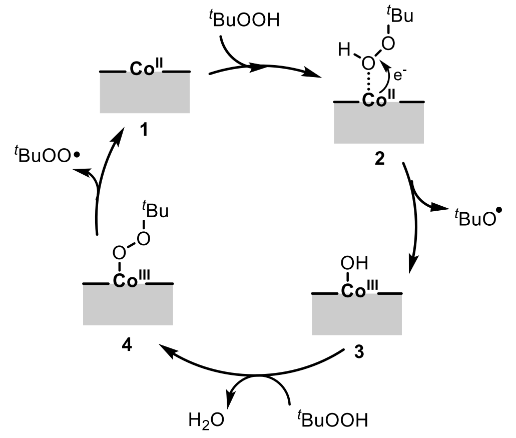

Scheme S1. Plausible activation pathway of ^{t}BuOOH by cobalt-based catalysts via the Haber-Weiss mechanism.[2,4,7]

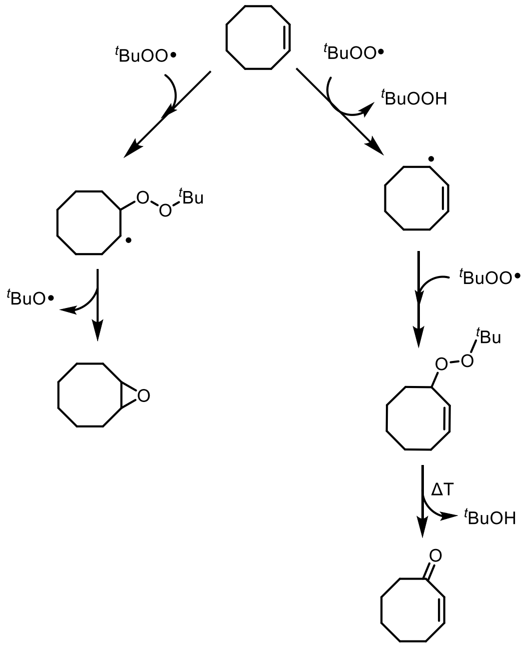

Scheme S2. Plausible mechanism for the (ep)oxidation of cyclooctene by freely diffusing ^{t}BuOO· (and/or ^{t}BuO·) radicals.[2,4,7]

25 References

1. C. Wegeberg, C. G. Frankær and C. J. McKenzie, *Dalton Trans.*, 2016, **45**, 17714-17722.
2. J. D. Koola and J. K. Kochi, *J. Org. Chem.*, 1987, **52**, 4545-4553.
3. J. Adam and R. Přibil, *Talanta*, 1971, **18**, 733-737.
4. M. Tonigold, Y. Lu, A. Mavrandonakis, A. Puls, R. Staudt, J. Möllmer, J. Sauer and D. Volkmer, *Chem. Eur. J.*, 2011, **17**, 8671-8695.
5. L. Saussine, E. Brazi, A. Robine, H. Mimoun, J. Fischer and R. Weiss, *J. Am. Chem. Soc.*, 1985, **107**, 3534-3540.
6. N. Turrà, U. Neuenschwander, A. Baiker, J. Peeters and I. Hermans, *Chem. Eur. J.*, 2010, **16**, 13226-13235.
7. U. Junghans, C. Suttkus, J. Lincke, D. Lässig, H. Krautscheid and R. Gläser, *Microporous Mesoporous Mater.*, 2015, **216**, 151-160.
